# Supplementary figures and images for: An ancestral transmembrane transcription factor couples cell envelope regulation and the SOS response in Caulobacter crescentus
Source: PLoS Genet. 2026 Jul 15;22(7):e1011986. doi: 10.1371/journal.pgen.1011986 (PMC13384398; doi:10.1371/journal.pgen.1011986)

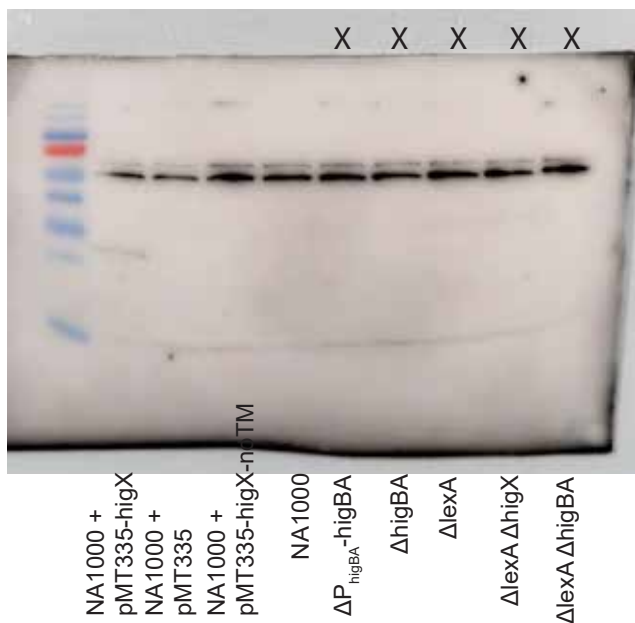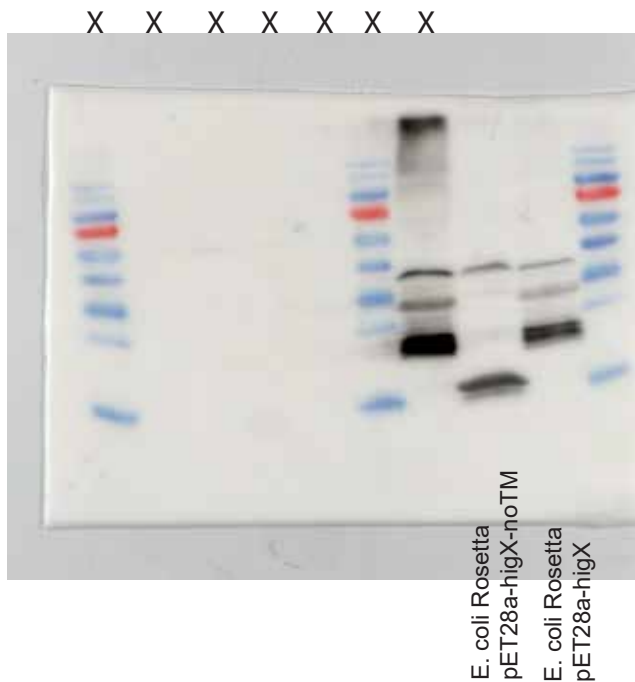

Supplement: S1 Raw Gel — (PDF) [file pgen.1011986.s004.pdf]

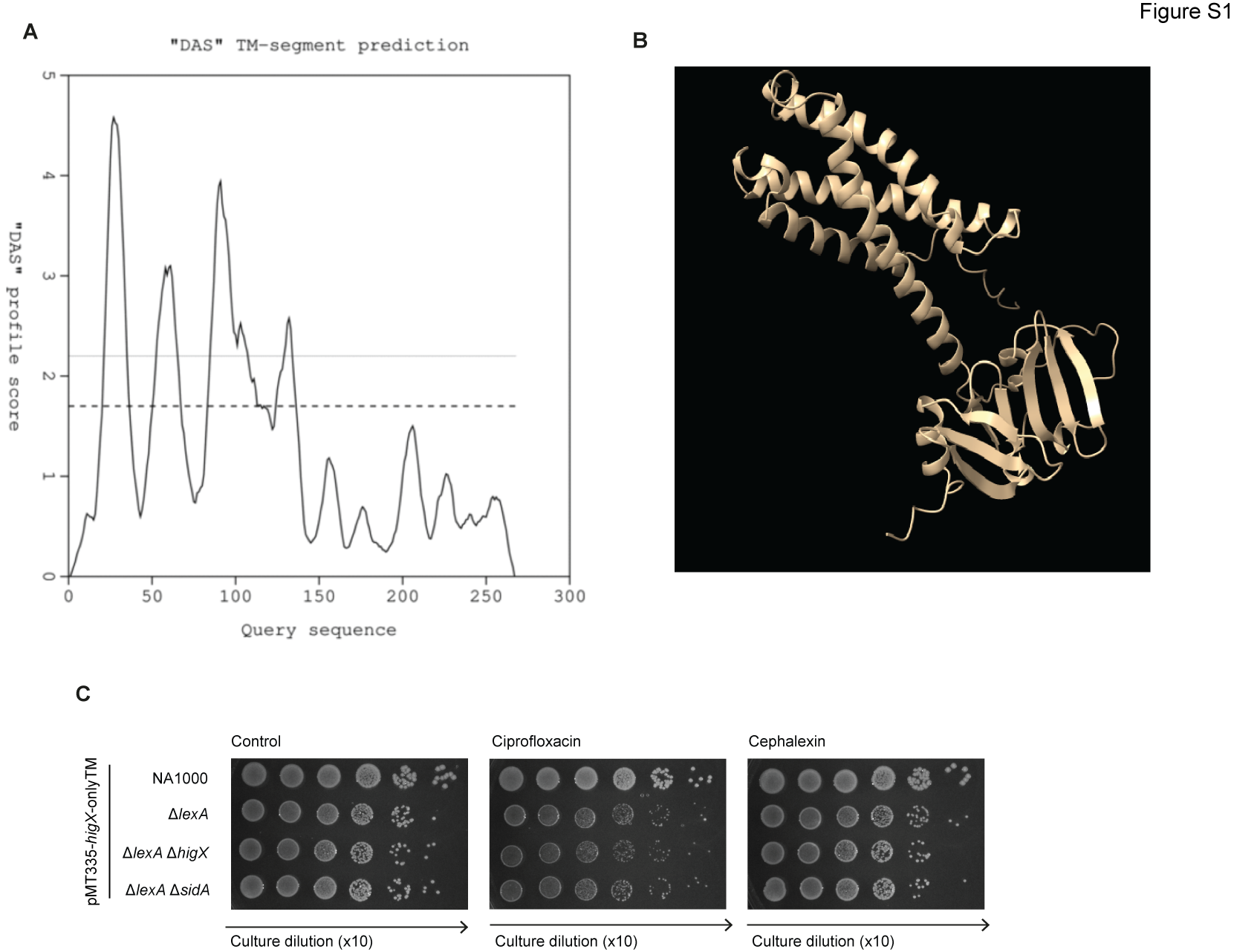

Supplement: S1 Fig — (A) Prediction of the transmembrane helix locations by DAS. (B) Model structure prediction of HigX by Alphafold, showing the N-terminal domain as a tightly packed four-helix bundle and the C-terminal domain with the typical LytTR DNA binding domain. (C) Efficiency of plating assay of WT, ΔlexA, ΔlexA ΔhigX and ΔlexA ΔsidA containing the truncated higX overexpression plasmid pMT335-higX-onlyTM on ciprofloxacin (0.5 μg/ml), cephalexin (5 μg/ml) or vehicle control. The empty vector control for this experiment is shown in Fig 4D. Images are representative of three independent biological replicates. (TIF) [file pgen.1011986.s006.tif]

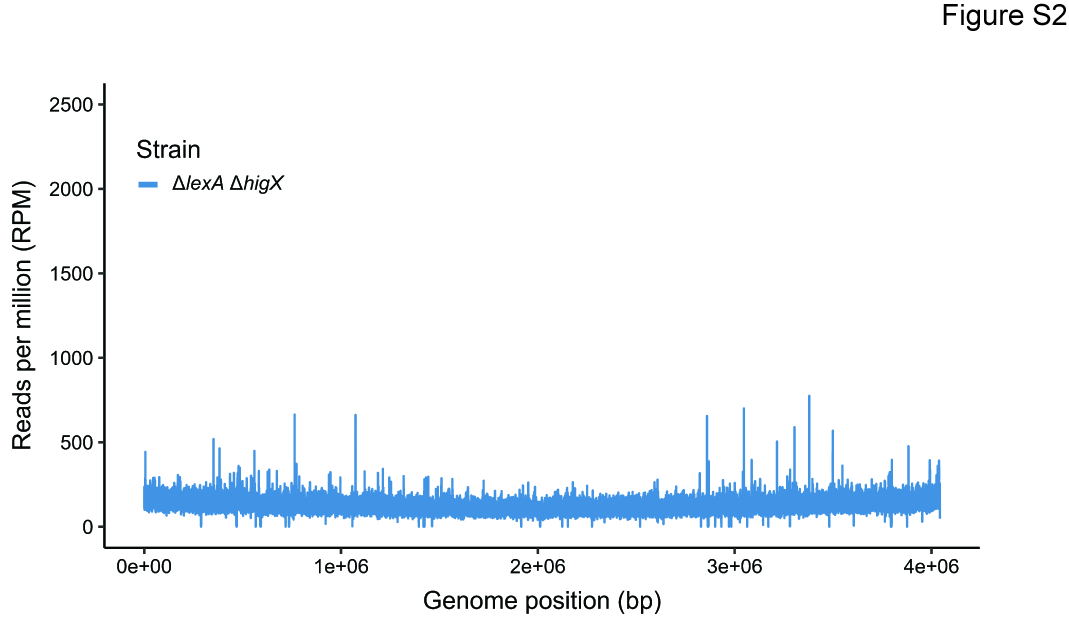

Supplement: S2 Fig — ChIP-seq profiles of HigX in the Caulobacter ΔlexA ΔhigX strain, with the data plotted as reads per million (RPM) across the genome. (TIF) [file pgen.1011986.s007.tif]

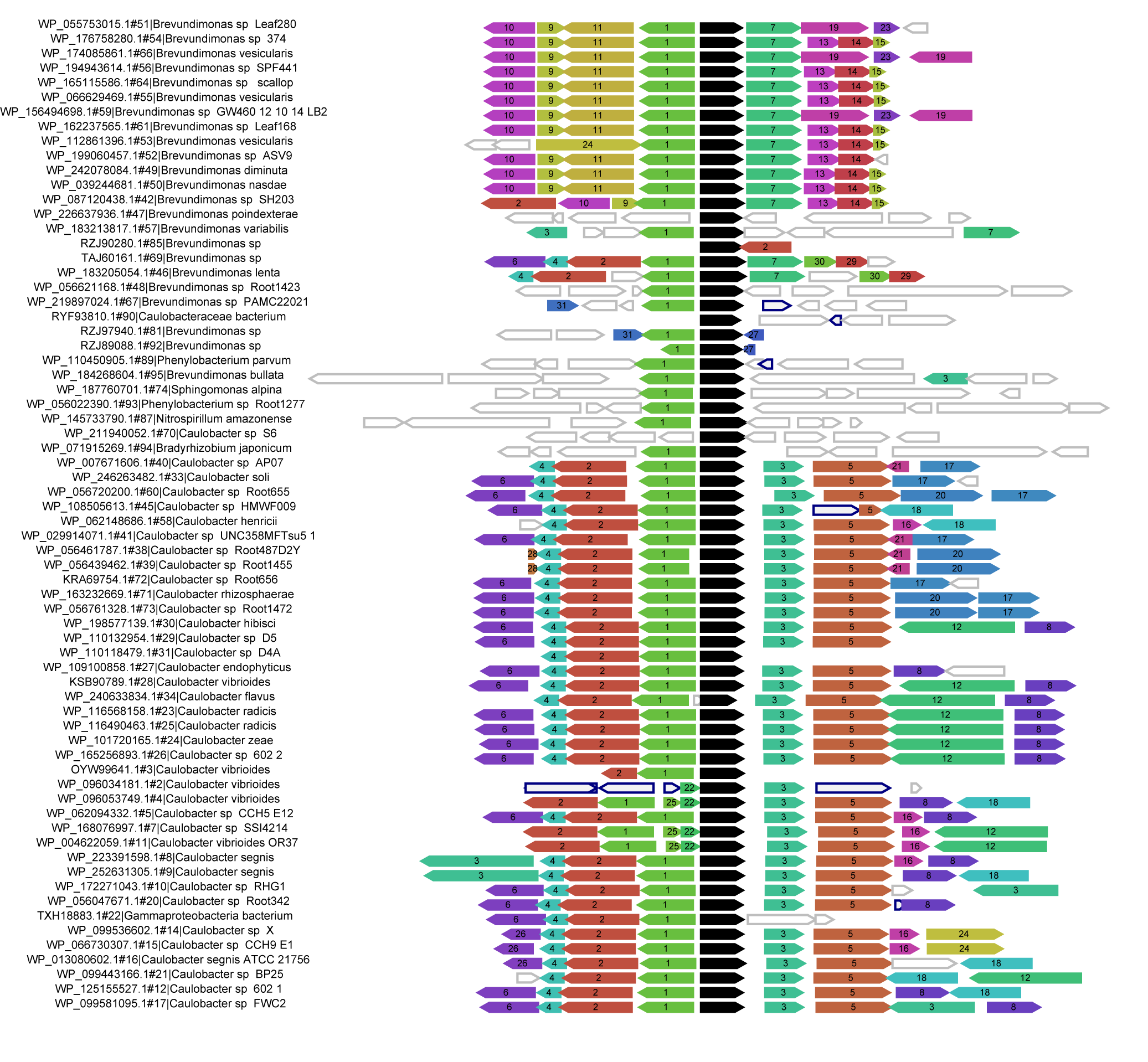

Supplement: S3 Fig — Output of FLaGs analysis using the results of a BLASTP search of HigX against known alpha-proteobacterial genomes (the BLASTP query sequence of Caulobacter crescentus NA1000 HigX is not included here, only the BLASTP output), with the limit of aligned genomic regions increased to 99, of which 68 were aligned. The number codes for selected proteins with annotated functions are as follows: 1, uncharacterized putative hydrolase; 2, FliI; 3, CtrA; 4, FliJ; 5, LdpD; 22, HigA; 25, HigB. (TIF) [file pgen.1011986.s008.tif]

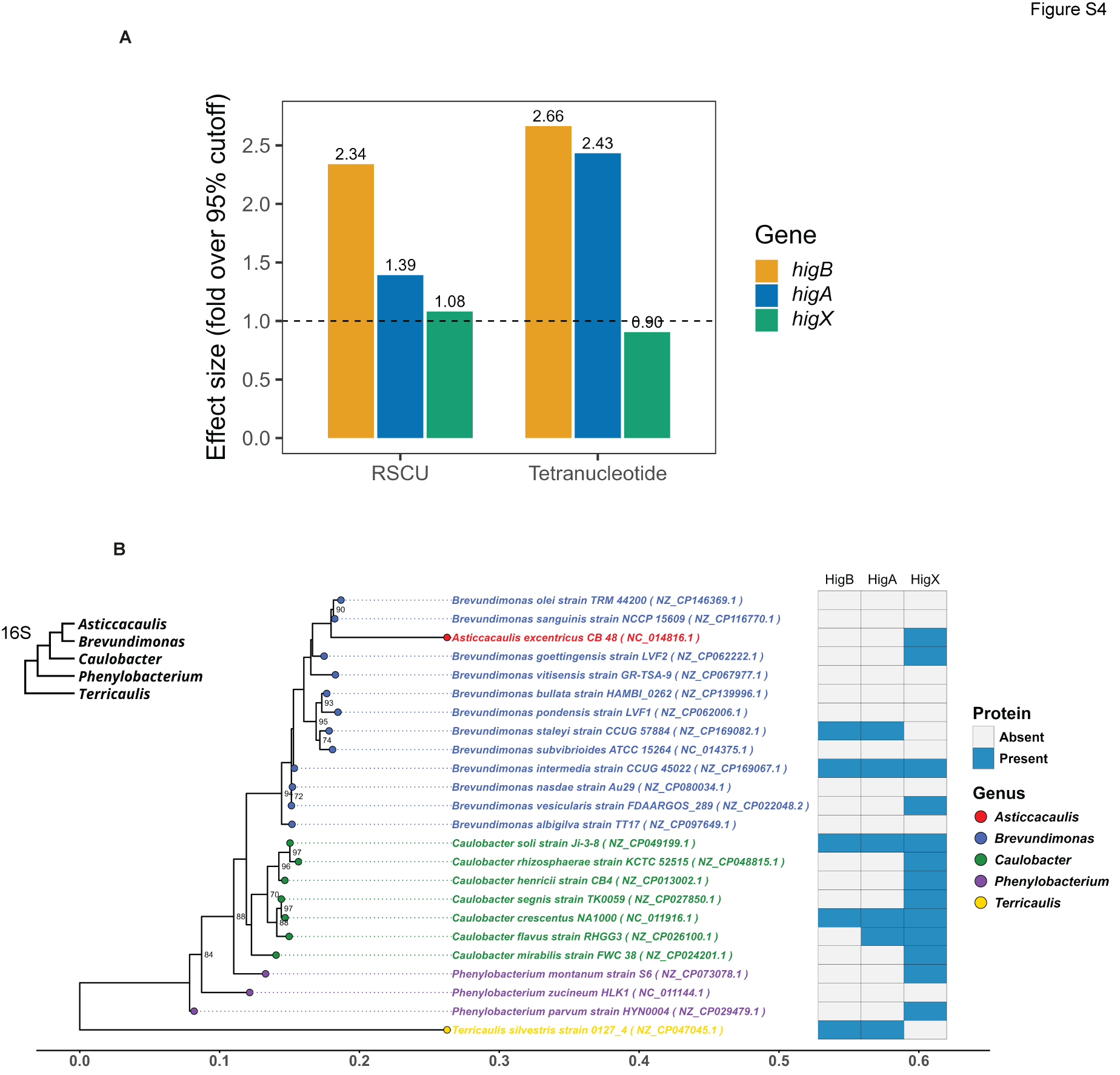

Supplement: S4 Fig — (A) Effect size analysis of higB, higA, and higX based on codon usage (RSCU) and tetranucleotide frequency composition. Bars represent the square root of the Mahalanobis distance2 (√MD2) relative to the 95% robust cutoff (MCD estimator), expressed as a fold over the threshold. The 95% cutoff (effect size = 1) is indicated as a dashed horizontal line, meaning that values above this indicate deviation from the genome-wide compositional background. (B) Phylogenetic tree of the order Caulobacterales showing the distribution of the proteins HigB, HigA, and HigX. The maximum-likelihood tree was constructed from 16S rRNA sequences of complete and reference genomes (n = 24) retrieved from NCBI, serving as a phylogenetic standard for comparison. The accompanying heatmap indicates the presence or absence of the three proteins across the same genomes. Note that the heatmap does not account for gene synteny. (TIF) [file pgen.1011986.s009.tif]

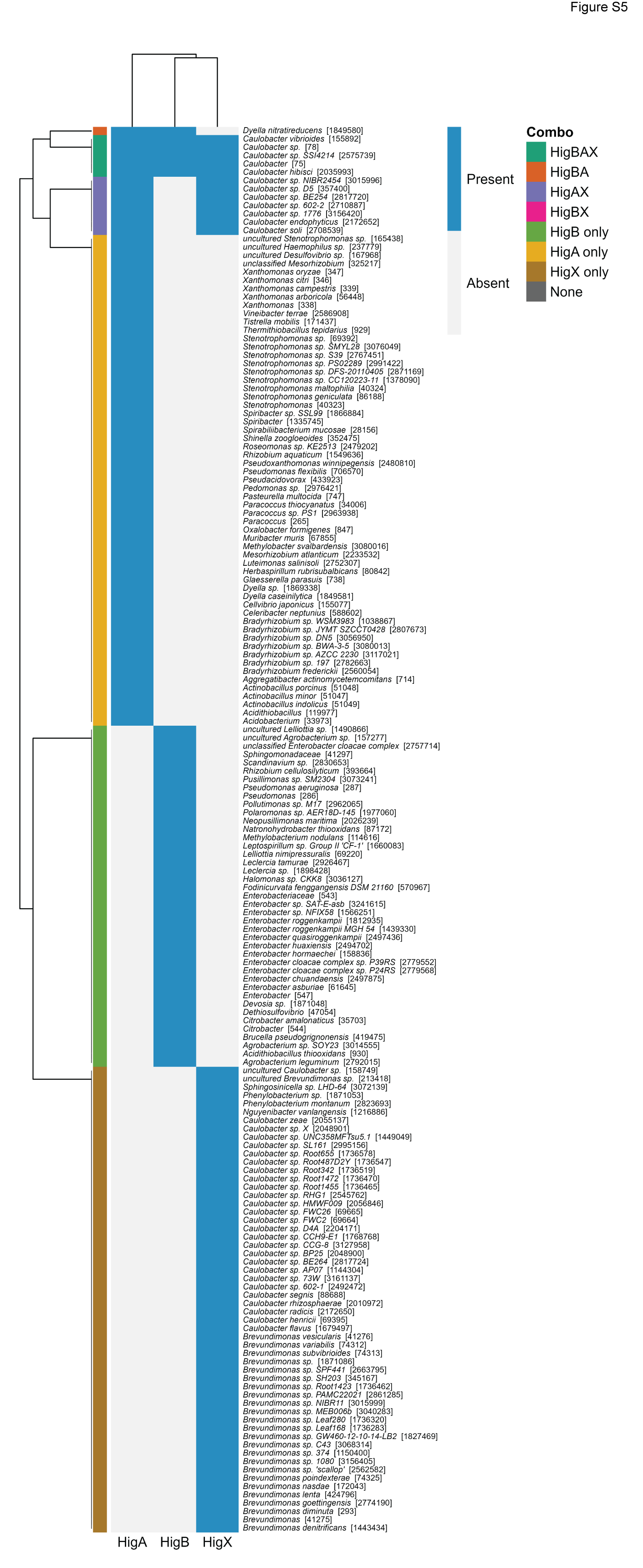

Supplement: S5 Fig — Presence–absence heatmap showing the occurrence of HigB, HigA, and HigX homologs across bacterial genomes identified by BLASTX searches against the NCBI RefSeq protein database. Each column indicates one of the three proteins, and each row corresponds to an organism with both its scientific name and NCBI TaxID. Presence/absence (blue/grey) is indicated by the left sidebar, and the right sidebar indicates protein combinations. Note that the heatmap does not account for gene synteny. (TIF) [file pgen.1011986.s010.tif]

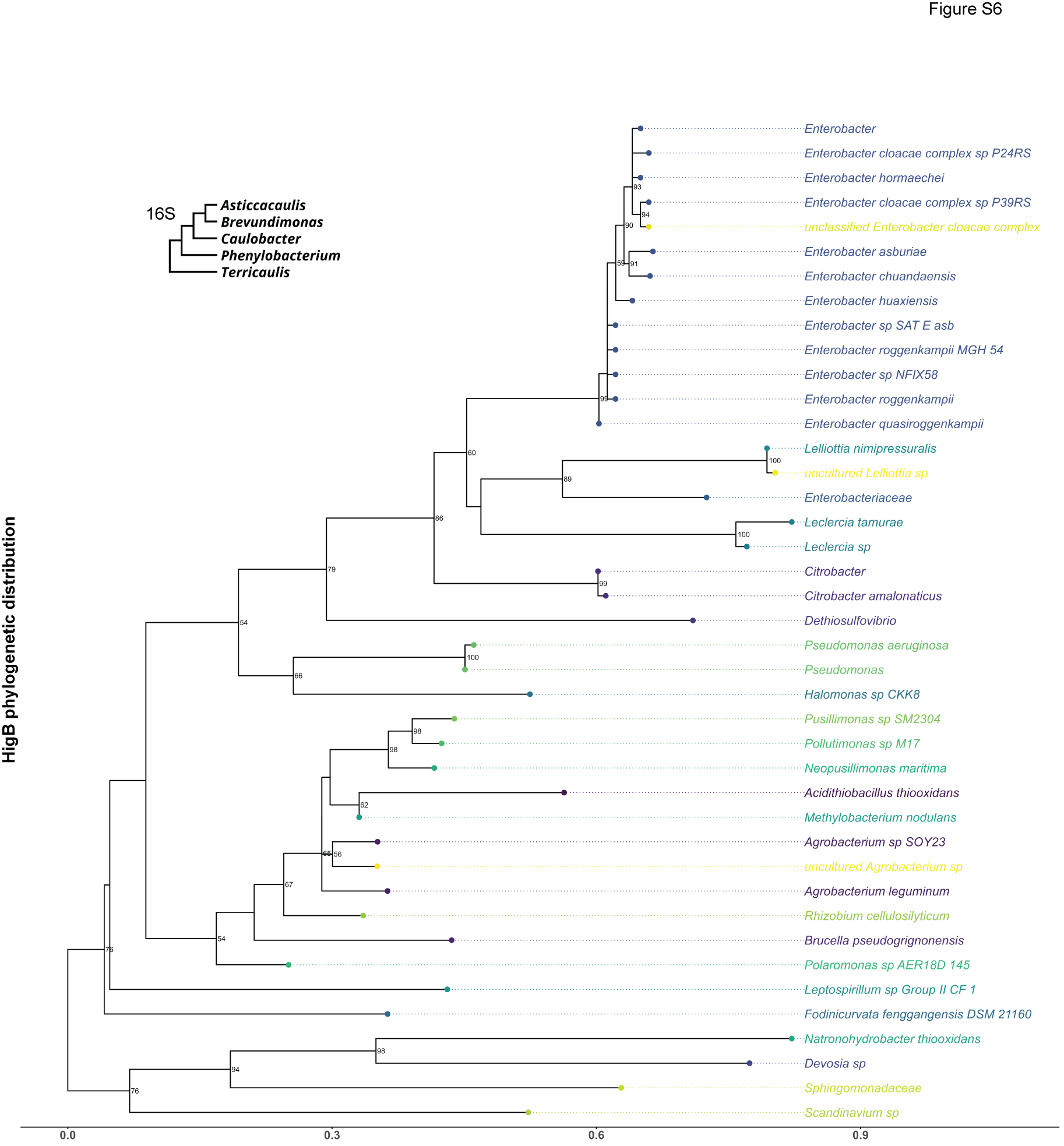

Supplement: S6 Fig — Phylogenetic tree of HigB homologs based on protein sequences retrieved from the top BLASTX hits. Sequences were aligned with MAFFT (L-INS-i), followed by inference of the tree with IQ-TREE using the maximum-likelihood method. The tree was midpoint-rooted for visualization, with tip labels displaying organism names, which have been colored by genus. Bootstrap support values (≥ 50) are displayed at internal nodes, and the scale bar indicates the number of amino-acid substitutions per site. The phylogram in the upper left corner shows a condensed version of the 16S phylogenetic tree (S4B Fig), which acts as a phylogenetic standard for comparison. (TIF) [file pgen.1011986.s011.tif]

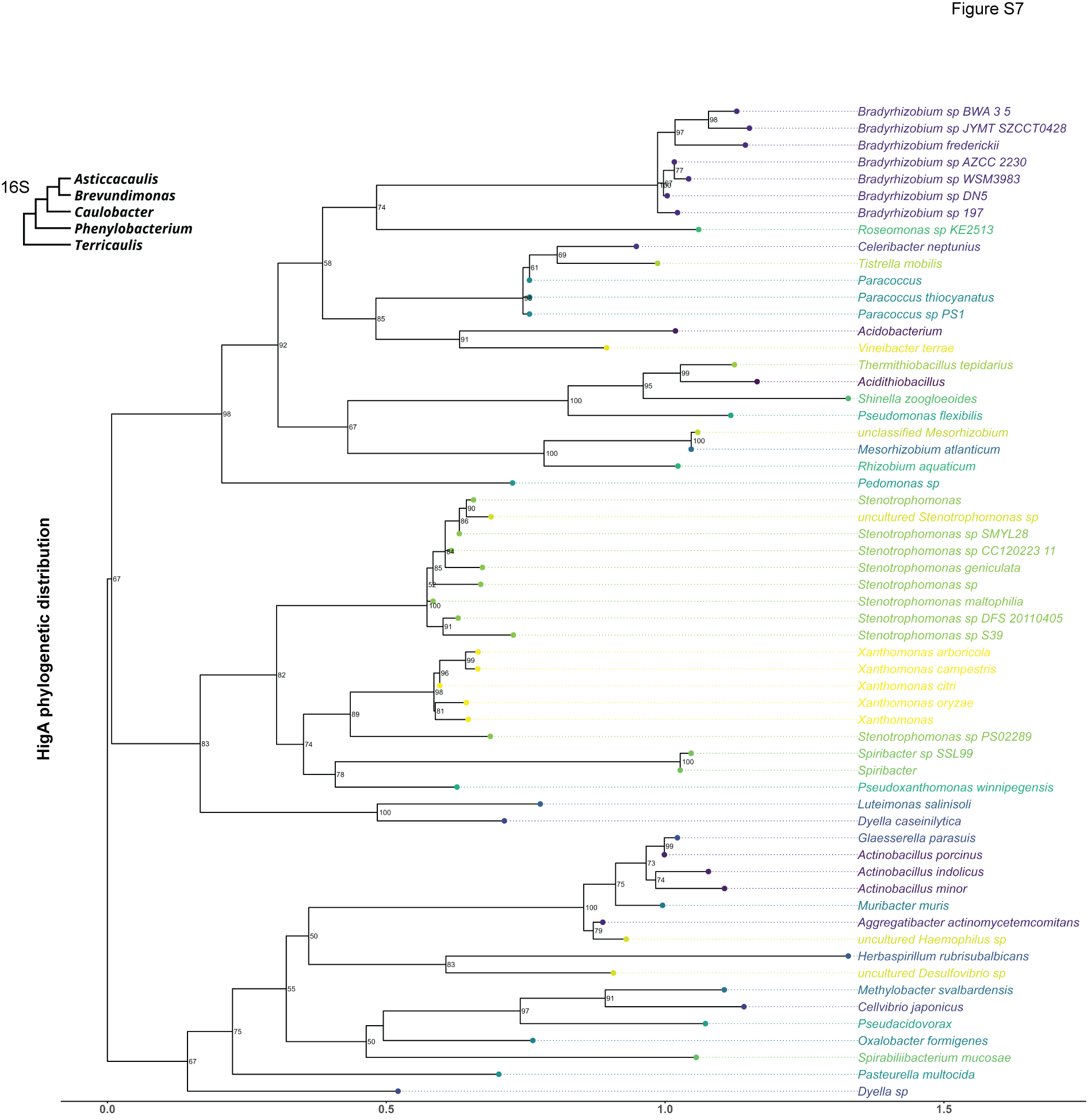

Supplement: S7 Fig — Phylogenetic tree of HigA homologs based on protein sequences retrieved from the top BLASTX hits. Sequences were aligned with MAFFT (L-INS-i), followed by inference of the tree with IQ-TREE using the maximum-likelihood method. The tree was midpoint-rooted for visualization, with tip labels displaying organism names, which have been colored by genus. Bootstrap support values (≥ 50) are displayed at internal nodes, and the scale bar indicates the number of amino-acid substitutions per site. The phylogram in the upper left corner shows a condensed version of the 16S phylogenetic tree (S4B Fig), which acts as a phylogenetic standard for comparison. (TIF) [file pgen.1011986.s012.tif]

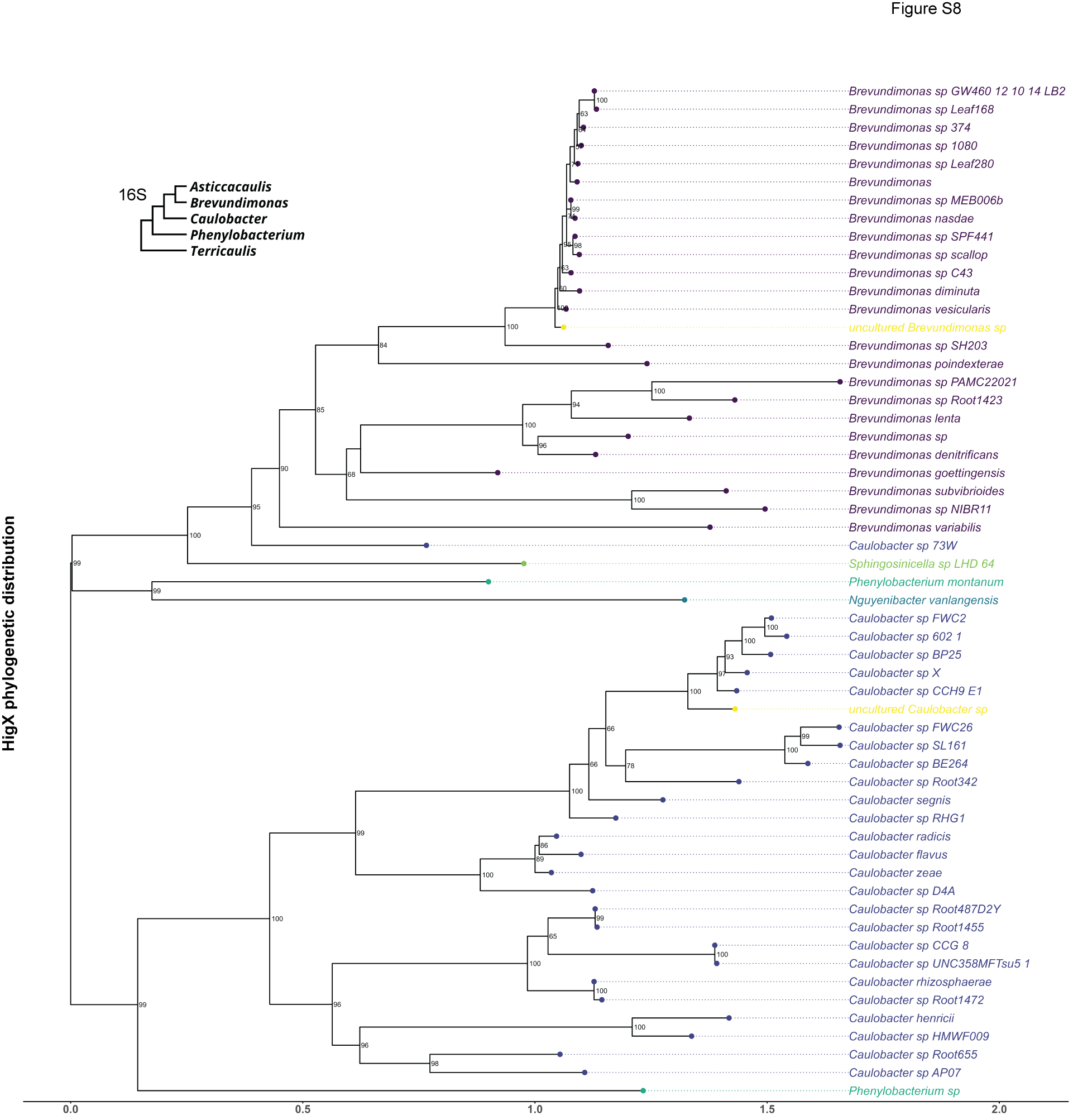

Supplement: S8 Fig — Phylogenetic tree of HigX homologs based on protein sequences retrieved from the top BLASTX hits. Sequences were aligned with MAFFT (L-INS-i), followed by inference of the tree with IQ-TREE using the maximum-likelihood method. The tree was midpoint-rooted for visualization, with tip labels displaying organism names, which have been colored by genus. Bootstrap support values (≥ 50) are displayed at internal nodes, and the scale bar indicates the number of amino-acid substitutions per site. The phylogram in the upper left corner shows a condensed version of the 16S phylogenetic tree (full version in S4B Fig), which acts as a phylogenetic standard for comparison. (TIF) [file pgen.1011986.s013.tif]
