# Supplementary material for: An ancestral transmembrane transcription factor couples cell envelope regulation and the SOS response in Caulobacter crescentus
Source: PLoS Genet. 2026 Jul 15;22(7):e1011986. doi: 10.1371/journal.pgen.1011986 (PMC13384398; doi:10.1371/journal.pgen.1011986)
Supplement: Attachment — Submitted filename: Brejndaletal2025.docx [file pgen.1011986.s014.docx]

**Summary:**

The manuscript by Brejndal et al. investigates an intriguing gene downstream of the *higBA* toxin-antitoxin pair that is involved in antibiotic resistance. The study stems from the confusing observation that deletion of *higBA* leads to a different phenotype than *higB* deletion. Through deletion and overexpression analysis, they find that the downstream *higX* gene is likely responsible for this drug resistance phenotype. In particular, they observe that HigX levels increase in *∆lexA* cells and that this increase contributes to antibiotic sensitivity. Through ChIP-seq experiments, the authors also find that the HigX transcription factor targets a variety of envelope-related and metabolic genes, and that this targeting appears impaired in the *∆lexA* background. Their genetic experiments and phylogenetic analyses also suggest that HigX acts independently of HigBA. Ultimately, the authors present a model in which elevated HigX levels may destabilize the cell envelope, ultimately leading to increased antibiotic sensitivity. Overall, I found the experimental logic to be sound and the manuscript to be well-written. Due to the variety of genetic strains and several screens, the interpretation is often complicated but presented clearly. However, I have concerns about the ChIP-Seq methodology and have suggestions for minor clarifications in the figures/text.

**Major points:**

- Can the authors justify the lack of input or mock controls in their ChIP-Seq methodology (unless I misread)? How does one know if peaks are real or simply recovery biases or sequencing artifacts (especially for smaller peaks)? The *∆lexA* sample may control for this issue, but it’s hard to say given the global effects of that mutation and apparent lower background. Alternatively, do they have EMSA data for a less-enriched target that validates the approach?

**Minor points:**

- Fig. 2: Can the authors clarify the 2A model figure? As drawn, it suggests that the *higB* transcriptional fusion has great overlap with the *higB* ORF than the translational fusion.
- Fig. 2: Given that the TSS for *higBA* is slightly upstream of *higB*, does a transcriptional fusion that includes all regions upstream of the ORF behave similarly to the original transcriptional fusion? To be clear, I’m not suggesting that this experiment is required, but I’m curious if the authors tried this.
- Line 202-204: I would be careful with this statement. Since this experiment compares overexpression of a transmembrane and cytoplasmic construct, there are many factors that could be at play, including level of overexpression, stability, polyclonal antibody sensitivity, and even stability of the membrane protein in sample buffer. Thus, I might amend to say that it’s clear that the antibody can detect the truncated construct (without trying to extrapolate stability arguments).
- Line 228: The authors may want to tighten the language here. As they note near the end of the paragraph, panel B indicates that HigX is partially responsible for the ciprofloxacin sensitivity in the *∆lexA* strain. But in other sentences they leave out “partially”, creating some confusion. To me, panel A indicates that HigX overproduction reduces viability upon ciprofloxacin treatment but does not necessarily directly demonstrate that it is responsible for the sensitivity of the *∆lexA* strain.
- The authors’ model suggests that reduced HigX levels may be responsible for the ciprofloxacin resistance of the *∆lexA* *∆higB* strain. I may have missed this, but have they tested to see if HigX levels are decreased in this strain relative to *∆lexA*?
- Lines 326-327: I’m not requesting a new experiment, but I’m curious if the authors have examined these strains via microscopy for any obvious envelope defects.
