# Supplementary material for: An ancestral transmembrane transcription factor couples cell envelope regulation and the SOS response in Caulobacter crescentus
Source: PLoS Genet. 2026 Jul 15;22(7):e1011986. doi: 10.1371/journal.pgen.1011986 (PMC13384398; doi:10.1371/journal.pgen.1011986)
Supplement: Attachment — Submitted filename: PLOS Genetics 2025 Brejndal.docx [file pgen.1011986.s015.docx]

PLOS Genetics 2025 Brejndal et al :

**Summary:**

In this manuscript, the authors identify HigX as a previously uncharacterized transmembrane transcription factor involved in maintaining cell envelope integrity in certain members of the Caulobacterales. This function was uncovered in a specific genetic context, namely a Caulobacter crescentus strain lacking both the HigBA toxin-antitoxin system and the LexA SOS-response regulator. The ∆lexA ∆higBA mutant displays increased sensitivity to ciprofloxacin, a phenotype that is reversed by deletion of higX. In C. crescentus, higX is located immediately downstream of higBA. Bioinformatic analyses indicate that higBA and its LexA-regulated promoter were likely acquired recently in some Caulobacter species via horizontal gene transfer. ChIP-seq analysis identified HigX binding sites across the chromosome and defined a consensus DNA-binding motif. In addition, forward genetic screens identified transposon mutants that either restore ciprofloxacin resistance to the ∆lexA ∆higBA strain or increase its tolerance to higX overexpression. All four identified insertions mapped to genes encoding proteins associated with the cell envelope, further supporting a role for HigX in envelope homeostasis.

This manuscript presents a substantial amount of experimental data. However, although HigX is conserved across some Caulobacterales, its regulation by the SOS response appears to be restricted to a limited subset of Caulobacter and Brevundimonas species. Based on the results presented, the proposal that HigX functions as a novel factor involved in cell envelope regulation remains largely hypothetical.This hypothesis would be more convincingly supported by investigating HigX function in a species that naturally lacks the higBA loci and by confirming direct regulation of genes involved in cell envelope or carbohydrate transport.

To broaden the appeal of the manuscript and strengthen the central conclusions, additional experimental support would be valuable, for example:

- Assess the phenotypic consequences of higX deletion or overexpression in other species that do not harbor higBA.
- Perform heterologous complementation experiments using higX genes from species lacking the higBA loci.
- Validate the regulatory activity of HigX on selected putative target genes.

**Major concerns:**

1. The presence of a ChIP-seq peak does not necessarily imply transcriptional regulation. Have you assessed whether the expression of putative target genes is altered upon higX overexpression or deletion, for example by β-galactosidase reporter assays or qRT-PCR? In addition, have any of the ChIP-seq peaks been independently validated by qChIP? Could HigX help organize the chromosome in the cell instead of playing a regulatory role?
2. Have you attempted heterologous complementation using higX homologs from other species, particularly from species that do not encode the higBA toxin-antitoxin system?
3. **Forward genetic screens:** Have you tested whether the ciprofloxacin sensitivity of the ∆lexA ∆higBA strain can be restored by complementation of the individual transposon mutants with the corresponding genes (CCNA_03248, CCNA_00669, and amiC)? Do you have a working hypothesis to explain how these genes that affect a distinct component of the cell envelope (TonB-dependent receptor in the outer membrane, LPS, and peptidoglycan, respectively), might alleviate ciprofloxacin sensitivity?

**Minor comments:**

Line 154: The pMT335-*higX* plasmid could not be transformed in the ∆*lexA* ∆*higBA* strain but have you tried to complement the quadruple mutant ∆*lexA* ∆*higBAX*?

Line 148: remove “of”

Line 169: remove the comma

Line 277-285: Could it be a general increase in cell envelope permeability? Have you tried other families of antibiotics (aminoglycosides) or big molecules that are not usually used against Gram negative bacteria (vancomycin)?

Lines 322-326: Have you tried to overexpress another LytTR proteins in the ∆*lexA* strain? Could this be as deleterious as *higX* overexpression?

Line 590: rpoDfod instead of rpoDfwd?

In the source data (excel file), *higX* is still annotated as *higC*
