# Supplementary material for: An ancestral transmembrane transcription factor couples cell envelope regulation and the SOS response in Caulobacter crescentus
Source: PLoS Genet. 2026 Jul 15;22(7):e1011986. doi: 10.1371/journal.pgen.1011986 (PMC13384398; doi:10.1371/journal.pgen.1011986)
Supplement: Attachment — Submitted filename: Response to Reviewers.docx [file pgen.1011986.s017.docx]

PGENETICS-D-25-01322
An ancestral transmembrane transcription factor couples cell envelope regulation and the SOS response in Caulobacter crescentus
PLOS Genetics

Dear Editors,

We have revised our manuscript according to the reviewer feedback and present the point-by-point responses to specific comments below (author answers in blue). We believe we have addressed all the reviewer comments, and have focused particularly on the phenotypic experiments by performing EOP assays with (1) *higX* overexpression in other filamentous mutants and in the non-filamentous Δ*lexA* Δ*sidA* mutant, (2) overexpression of heterologous *higX* variants from other species and (3) overexpression of a *higX*-onlyTM variant. These have allowed us to show that (1) the *higX* overexpression phenotype is not dependent on *lexA* but on the degree of filamentation of the cells, (2) HigX function is partially but not fully conserved between species, and (3) the effect is not due to HigX protein overload in the membrane since an onlyTM truncated construct was not toxic, but instead protective.

We have also performed beta-galactosidase assays on selected HigX ChIP-Seq target promoters, and complementation experiments with the genes isolated as suppressors by the forward genetic screening. We have addressed the major concern of reviewer 2 regarding the lack of ChIP-Seq control by including the plot from a genetic negative control experiment that we had performed but not included in the first version, namely the Δ*lexA* Δ*higX* strain. In the absence of *higX*, we only see background noise. Overall, the new experiments we have performed are generally supportive of the manuscript’s main hypothesis and extend the conceptual advance of the work further by showing that it is cell filamentation generally which sensitises to higX overexpression (and ciprofloxacin), independently of presence or absence of LexA.

We thank all reviewers for their helpful and constructive feedback and hope that this revised version will be found suitable for publication. I furthermore confirm that the vector art of Figure 6A was hand-drawn by me in Adobe Illustrator and can provide the original source file if requested.

Sincerely,

Clare Kirkpatrick

**Response to reviewer comments**

Reviewer #1:

In this manuscript, the authors characterised the enigmatic membrane-associated transcription factor called HigX in Caulobacter crescentus. In C. crescentus, higX is encoded downstream of the TA system higBA, whose expression is repressed by LexA and the HigA antitoxin. While a previous study showed that ∆lexA cells display a HigB-dependent hypersensitivity to ciprofloxacin, the authors show in this manuscript that the concomitant inactivation of both repressors LexA and HigA leads to a HigB-independent hypersensitivity to ciprofloxacin, which depends on HigX. The expression of higX from an inducible promoter on a multi-copy plasmid also leads to ciprofloxacin hypersensitivity but only in a ∆lexA background. Interestingly, ChIP-seq experiments show that HigX binds to promoter regions of cell envelope-associated genes and that DNA binding of HigX requires LexA. In addition, mutations in cell envelope-related genes were found in forward genetic screens to suppress HigX-dependent sensitivity to ciprofloxacin in a ∆lexA ∆higBA background, suggesting that overproduction of HigX leads to destabilization of the cell envelope. Finally, a thorough phylogenetic analysis and RSCU of the hig locus show that higX is widespread in Caulobacterales whereas higBA was likely acquired by horizontal gene transfer in the Caulobacter genus.

Although the phenotypes displayed by HigX overexpression are interesting, they are only observed in ∆lexA backgrounds and upon antibiotics treatment, limiting the understanding of its biological role.

In particular, we don’t know why the phenotypes are observed only in ∆lexA, even when higX is overproduced from an inducible promoter on a multicopy plasmid. In fact, it is unclear if the toxicity of HigX in ∆lexA cells exposed to ciprofloxacin comes from transcriptional misregulation, since HigX cannot bind anymore to DNA in the absence of lexA, or from the (over)accumulation of HigX in the membrane of filamentous cells.

I would therefore strongly encourage the authors to further characterise the dependency of the HigX-dependent phenotypes on LexA.

Here are suggestions to address this question:

1 - The authors proposed that the extreme filamentation of ∆lexA cells leads to excessive accumulation of HigX in the cell envelope, which in turn weakens its integrity and facilitates access of ciprofloxacin to the cytoplasm. If this is true, inactivating sidA in ∆lexA should decreased ciprofloxacin sensitivity upon higX overexpression since ∆lexA∆sidA cells do not filament (DOI: 10.1101/gad.2038911).

We have performed these experiments and present the data in new Figure panels 4D and 5G. Consistent with the hypothesis, the non-filamentous ΔlexA ΔsidA mutant was indeed less sensitive than the ΔlexA parent strain, both to HigX overexpression, ciprofloxacin and cephalexin. It was not 100% protected from ciprofloxacin, but this is likely due to the negative effects of constitutive SOS response activation in this strain.

2 - On the other hand, viable or conditional mutants that generate filamentous cells could be used to test whether cellular filamentation indeed sensitises to ciprofloxacin or cephalexin upon higX overexpression. There are indeed numerous filamentous cell division or cell cycle mutants available in Caulobacter.

We have performed these experiments and present the data in new Figure 4C, using the cell cycle mutants Δ*tipN*, Δ*lon* and Δ*popZ* which develop filamentous cells to varying degrees (Δ*tipN* the least severe, Δ*popZ* the most severe). Noticeably, these cells were extremely sensitized to ciprofloxacin (even though they have intact SOS response) and to HigX overexpression, both separately and together. Qualitatively, the degree of sensitivity even seemed to correlate with the degree of severity of filamentation (WT < Δ*tipN* < Δ*lon* < Δ*popZ*). From this result and the experiments with the Δ*lexA* Δ*sidA* double mutant (see previous comment) we are confident that we can develop the model proposed in this paper further, to ascribe the HigX-dependent phenotypes to filamentous cells and not exclusively to LexA.

3 - As a complementary approach to the higX-noTM variant, a higX-onlyTM could be expressed from the pMT335 plasmid vector to test if the TM domains alone can induce the same hypersensitivity to ciprofloxacin. If it works, it would support the hypothesis of cell envelope loading.

We have made this construct by truncating HigX at aa143 and cloning into pMT335, performed this experiment and present the results in new figure S1C. Here, we were surprised to see that the transmembrane-only construct, far from being toxic, was actually protective! Since it was not toxic, we cannot conclude that cell envelope overload is the cause of the phenotypes, and have toned this aspect down in the text, since the original and new data together now support that altered gene regulation by HigX is more likely to be responsible. We currently have no hypothesis for why HigX-onlyTM should be actively protective, unless it is by stabilizing the envelope in some unknown way. We will be following this up in future work.

4 - To test whether DNA binding of HigX is responsible for the phenotypes, the HigX-dependent regulon should be determined by ChIP-seq (and RNA-seq) in cells exposed to ciprofloxacin. Indeed, the ChIP-seq has been done in PYE, a condition in which neither the lexA nor the hig mutants impact growth. To avoid cell death with ciprofloxacin, one of the suppressors could be used. For instance, WT, ∆lexA ∆higBA, ∆lexA ∆higBA 03248::himar1 and ∆lexA ∆higBAX strains could be grown in the presence of the antibiotic before performing the ChIP-seq or extracting RNA.

If the first three experiments support the first hypothesis, there is even no need to address the second hypothesis.

Since we have obtained a large amount of new data that is mostly supportive of the main hypothesis from the first three proposed experiments and the others we have done in response to Reviewer 2 and Reviewer 3’s suggestions, and from our previous work (reference 15) where we showed that the activating effect of ciprofloxacin on the LexA-dependent *higBA* promoter was not very strong, we have not performed this specific experiment.

However, although it is out of the scope of this manuscript, we have generated quantitative proteomics datasets from follow-up mass spectrometry experiments (separately from the data in Figure 3E) for higX overexpression and higX deletion, and have found many occurrences of proteins whose abundances are significantly changed, and whose genes have HigX binding peaks in the ChIP-Seq data. The majority of these are in cell envelope and energy generation COG groups, similarly to the ChIP peak classification in Figure 5C. From this additional data, which is being written up for a separate manuscript, we are reasonably confident that HigX DNA binding is likely responsible for the phenotypes.

Minor comments

- L223: It is stated that the abundance of HigX peptides was “strongly depleted in ∆higX”.
Why HigX peptides are still detected in mass spectrometry in ∆higX cells?

This is on account of the TMT quantification method used, in which there is a small amount of co-isolation of peptides from other proteins in the window used for ion selection based on size. Because the TMT isotope labels are applied post-selection, these come through assigned as HigX peptides in the deletion strain. This is not a confounding factor in the calculations of abundance ratios in the other strains where HigX is intact, because these peptides would cancel each other out. The deletion of *higX* was confirmed by colony PCR and sequencing before the mass spectrometry experiment, so we are confident that it is the correct strain.

- Do the suppressors also induce ciprofloxacin resistance to ∆lexA mutant? If so, this would mean that the suppressors also protect from HigB-dependent toxicity.

We agree that that would be consistent with the data but have not tried this experiment yet, since we only transduced the insertions into the Δ*lexA* Δ*higBA* background yet.

Reviewer #2:

**Summary:**

The manuscript by Brejndal et al. investigates an intriguing gene downstream of the *higBA* toxin-antitoxin pair that is involved in antibiotic resistance. The study stems from the confusing observation that deletion of *higBA* leads to a different phenotype than *higB* deletion. Through deletion and overexpression analysis, they find that the downstream *higX* gene is likely responsible for this drug resistance phenotype. In particular, they observe that HigX levels increase in *∆lexA* cells and that this increase contributes to antibiotic sensitivity. Through ChIP-seq experiments, the authors also find that the HigX transcription factor targets a variety of envelope-related and metabolic genes, and that this targeting appears impaired in the *∆lexA* background. Their genetic experiments and phylogenetic analyses also suggest that HigX acts independently of HigBA. Ultimately, the authors present a model in which elevated HigX levels may destabilize the cell envelope, ultimately leading to increased antibiotic sensitivity. Overall, I found the experimental logic to be sound and the manuscript to be well-written. Due to the variety of genetic strains and several screens, the interpretation is often complicated but presented clearly. However, I have concerns about the ChIP-Seq methodology and have suggestions for minor clarifications in the figures/text.

**Major points:**

- Can the authors justify the lack of input or mock controls in their ChIP-Seq methodology (unless I misread)? How does one know if peaks are real or simply recovery biases or sequencing artifacts (especially for smaller peaks)? The *∆lexA* sample may control for this issue, but it’s hard to say given the global effects of that mutation and apparent lower background. Alternatively, do they have EMSA data for a less-enriched target that validates the approach?

We had performed a parallel genetic negative control for the ChIP-Seq using the Δ*lexA* Δ*higX* strain. We had omitted to include it in the previous version. It is now shown in Supplementary Figure S2. It resembles the Δ*lexA* strain except with a higher level of background noise. This is due to much lower mapped sequencing read recovery in the negative control lacking HigX relative to the other strains (WT had 25 million reads, ΔlexA had 21.7 million reads, Δ*lexA* Δ*higX* had 469651 reads). Hence, the conversion to reads per million for normalization and comparison between datasets leads to a noisier baseline. Based on this data, and the positive beta-galactosidase reporter result for the relatively low-enriched peak of the CCNA_03248 promoter (new Fig 6E), we are reasonably confident that the peaks are not artefacts, and that HigX binding is reduced in ΔlexA but not totally eliminated.

**Minor points:**

- Fig. 2: Can the authors clarify the 2A model figure? As drawn, it suggests that the *higB* transcriptional fusion has great overlap with the *higB* ORF than the translational fusion.

This was a slight alignment error in the graphic, as it is drawn approximately but not exactly to scale. The translational fusion contains 10 codons/30 bp of the starting protein sequence of HigB, while the transcriptional fusion reporter (from our previous work, reference 15) has an overlap of 8 codons/24 bp (although these codons are not translationally fused to LacZ because in the plac290 vector the lacZ gene has its own SD sequence and start codon). We have aligned the red line better in the updated figure 2A.

- Fig. 2: Given that the TSS for *higBA* is slightly upstream of *higB*, does a transcriptional fusion that includes all regions upstream of the ORF behave similarly to the original transcriptional fusion? To be clear, I’m not suggesting that this experiment is required, but I’m curious if the authors tried this.

I am not sure which construct this reviewer is referring to as the “original transcriptional fusion” but we have presented in the paper all the transcriptional and translational data that we have obtained, apart from the negative control empty vector of the translational fusion, which was consistently zero to two Miller units.

- Line 202-204: I would be careful with this statement. Since this experiment compares overexpression of a transmembrane and cytoplasmic construct, there are many factors that could be at play, including level of overexpression, stability, polyclonal antibody sensitivity, and even stability of the membrane protein in sample buffer. Thus, I might amend to say that it’s clear that the antibody can detect the truncated construct (without trying to extrapolate stability arguments).

We agree that this would be better to phrase more conservatively and have amended it to “Therefore, we conclude that our antibody is capable of detecting both the full-length and the truncated HigX proteins, but that the abundance of the truncated variant in *Caulobacter* is below the detection limit under these experimental conditions.” (new lines 204-207)

- Line 228: The authors may want to tighten the language here. As they note near the end of the paragraph, panel B indicates that HigX is partially responsible for the ciprofloxacin sensitivity in the *∆lexA* strain. But in other sentences they leave out “partially”, creating some confusion. To me, panel A indicates that HigX overproduction reduces viability upon ciprofloxacin treatment but does not necessarily directly demonstrate that it is responsible for the sensitivity of the *∆lexA* strain.

We have amended “was” to “could be” in this line (new line 231) and amended “was responsible” to “was at least partially responsible” later in the paragraph (new line 238) in order to be more cautious at this point in the text.

- The authors’ model suggests that reduced HigX levels may be responsible for the ciprofloxacin resistance of the *∆lexA* *∆higB* strain. I may have missed this, but have they tested to see if HigX levels are decreased in this strain relative to *∆lexA*?

We agree that that would be consistent with the data but have not tried this experiment yet.

- Lines 326-327: I’m not requesting a new experiment, but I’m curious if the authors have examined these strains via microscopy for any obvious envelope defects.

By phase-contrast microscopy we have not seen any obvious differences in morphology between the Δ*lexA* single mutant and any of the double mutant derivatives (Δ*lexA* Δ*higBA*, Δ*lexA* Δ*higB* or Δ*lexA* Δ*higX*). They all grow as highly filamentous cells with very few constrictions, and none of them showed any noticeable increase in blebbing or visible lysis under normal growth conditions (although we have not examined them under antibiotic stress yet). Data on the growth kinetics and cell morphology of the Δ*lexA*, Δ*lexA* Δ*higB* and Δ*lexA* Δ*higBA* strains was included in our previous work (reference 15, in the supplementary material).

Reviewer #3:

**Summary:**

In this manuscript, the authors identify HigX as a previously uncharacterized transmembrane transcription factor involved in maintaining cell envelope integrity in certain members of the *Caulobacterales*. This function was uncovered in a specific genetic context, namely a *Caulobacter crescentus* strain lacking both the HigBA toxin-antitoxin system and the LexA SOS-response regulator. The ∆*lexA* ∆*higBA* mutant displays increased sensitivity to ciprofloxacin, a phenotype that is reversed by deletion of *higX*. In *C. crescentus*, *higX* is located immediately downstream of *higBA*. Bioinformatic analyses indicate that *higBA* and its LexA-regulated promoter were likely acquired recently in some *Caulobacter* species via horizontal gene transfer. ChIP-seq analysis identified HigX binding sites across the chromosome and defined a consensus DNA-binding motif. In addition, forward genetic screens identified transposon mutants that either restore ciprofloxacin resistance to the ∆*lexA* ∆*higBA* strain or increase its tolerance to *higX* overexpression. All four identified insertions mapped to genes encoding proteins associated with the cell envelope, further supporting a role for HigX in envelope homeostasis.

This manuscript presents a substantial amount of experimental data. However, although HigX is conserved across some Caulobacterales, its regulation by the SOS response appears to be restricted to a limited subset of *Caulobacter* and *Brevundimonas* species. Based on the results presented, the proposal that HigX functions as a novel factor involved in cell envelope regulation remains largely hypothetical.This hypothesis would be more convincingly supported by investigating HigX function in a species that naturally lacks the *higBA* loci and by confirming direct regulation of genes involved in cell envelope or carbohydrate transport.

To broaden the appeal of the manuscript and strengthen the central conclusions, additional experimental support would be valuable, for example:

- Assess the phenotypic consequences of *higX* deletion or overexpression in other species that do not harbor *higBA*.
- Perform heterologous complementation experiments using *higX* genes from species lacking the *higBA* loci.
- Validate the regulatory activity of HigX on selected putative target genes.

**Major concerns:**

1. The presence of a ChIP-seq peak does not necessarily imply transcriptional regulation. Have you assessed whether the expression of putative target genes is altered upon *higX* overexpression or deletion, for example by β-galactosidase reporter assays or qRT-PCR? In addition, have any of the ChIP-seq peaks been independently validated by qChIP? Could HigX help organize the chromosome in the cell instead of playing a regulatory role?

We have performed beta-galactosidase assays for selected target genes (*CCNA_03218*, *CCNA_03248*, *amiC*, see new Figures 5E and 6E) and found that so far CCNA_03248 is the only one for which we see altered activity upon *higX* overexpression in WT (in addition to the *higBA* promoter-reporter which we already showed was HigX-dependent in Figure 2C). However, due to the time constraints for the revisions, we have not been able to look at reporter activity in all genetic backgrounds that could be relevant.

Please see also the response to major comment 4 of Reviewer 1 regarding our ongoing quantitative proteomics experiments, which support the model that HigX DNA binding is regulatory rather than structural.

2. Have you attempted heterologous complementation using *higX* homologs from other species, particularly from species that do not encode the *higBA* toxin-antitoxin system?

We have now done heterologous complementation with two *higX* relatives (from *Caulobacter segnis* and *Brevundimonas lenta*, neither of which have *higBA* at the same locus) and present the results in new Figure 7D. The *Caulobacter segnis* homolog reduced growth without ciprofloxacin, complementing similarly to native higX, but did not sensitise cells to ciprofloxacin in the same way. The *Brevundimonas* gene did not complement at all, with or without ciprofloxacin. Therefore, some functional change seems to have occurred between species, although we don’t have enough data to propose a model yet.

3. **Forward genetic screens:** Have you tested whether the ciprofloxacin sensitivity of the ∆*lexA* ∆*higBA* strain can be restored by complementation of the individual transposon mutants with the corresponding genes (*CCNA_03248, CCNA_00669,* and *amiC*)? Do you have a working hypothesis to explain how these genes that affect a distinct component of the cell envelope (TonB-dependent receptor in the outer membrane, LPS, and peptidoglycan, respectively), might alleviate ciprofloxacin sensitivity?

We have done direct complementation of the CCNA_03248 gene, and overexpression experiments with amiC (since this is not a disruption suppressor), as well as beta-galactosidase assays for both their promoters, and present the results in new figure 6E, F and G. We show that amiC overexpression is protective to a similar degree as the PamiC insertion suppressor. CCNA_03248 overexpression from pMT335 was ambiguous, because the empty vector control for this experiment was sensitive to Cipro, unlike the resistant Tn insertion parent strain.

Since the amiC overexpression data was strongest, we are limiting ourselves to suggesting a model where increasing AmiC levels promotes successful cell division events and reduces filamentation (at the population level), which improves resistance. We have expanded on this in the text (new lines 374-381, 540-545).

**Minor comments:**

Line 154: The pMT335-*higX* plasmid could not be transformed in the ∆*lexA* ∆*higBA* strain but have you tried to complement the quadruple mutant ∆*lexA* ∆*higBAX*?

We did try, and could not transform this strain with pMT335-higX either. Presumably the high copy number of pMT335 derivatives in Caulobacter over-rides the lack of the chromosomal gene.

Line 148: remove “of”

Corrected

Line 169: remove the comma

Corrected

Line 277-285: Could it be a general increase in cell envelope permeability? Have you tried other families of antibiotics (aminoglycosides) or big molecules that are not usually used against Gram negative bacteria (vancomycin)?

We agree this is probably happening, and is likely the reason that the ΔlexA ΔhigBA CCNA_03248::himar1 mutant carrying the empty vector pMT335 becomes sensitive to ciprofloxacin even though it was supposed to be the control in this experiment, because there is gentamicin in these plates to maintain selection on the plasmid. This has been expanded on in the revised Discussion (new lines 530-545).

Lines 322-326: Have you tried to overexpress another LytTR proteins in the ∆*lexA* strain? Could this be as deleterious as *higX* overexpression?

We have now tried both heterologous higX from two other species, and higX-onlyTM, as previously described. See response to major comment 2, and to major comment 3 of Reviewer 1.

Line 590: rpoDfow instead of rpoDfwd?

This is intentional since the rpoD primer sequences were selected based on their use in another publication (da Rocha et al 2008, reference 19), and we used the original names.

In the source data (excel file), *higX* is still annotated as *higC*

Has been corrected (and the new source data for revision beta-gals in Figure 5 and Figure 6 has been added)
