# Supplementary material for: An ancestral transmembrane transcription factor couples cell envelope regulation and the SOS response in Caulobacter crescentus
Source: PLoS Genet. 2026 Jul 15;22(7):e1011986. doi: 10.1371/journal.pgen.1011986 (PMC13384398; doi:10.1371/journal.pgen.1011986)
Supplement: Attachment — Submitted filename: Response to Reviewers 2.docx [file pgen.1011986.s018.docx]

Dear Editors,

Thank you for the feedback on the revised manuscript and its new data. I have revised the text in all places requested and changed figures where needed. In particular, I have taken out the ambiguous complementation experiment of Figure 6F and changed the replicate images used for Figure 4D. For some reason the images used before had different brightness/contrast settings to all the others, which I think is the major reason for the reviewer concern. With the visual settings fixed, the plates are now more consistent with the observations of the other experiments and point to the same conclusions.

Please see below for the full response to editor and reviewer comments. I hope that this version will be considered acceptable. Thanks again for the input on the manuscript.

Best regards,

Clare

***Response to Editors***

Thank you for the major revisions and additional work in your manuscript regarding the function of higX. The initial concerns of the reviewers were largely satisfied. The new data with other filaments mutants and with the ∆lexA ∆sidA strain nicely strengthens this work by clarifying models about higX activity. However, some concerns were raised about the new data that require minor revisions.

Multiple reviews raised concerns about the variability in the spot dilution assays presented, particularly in 4D. In addition, the images in 4D seem poorly illuminated and difficult to interpret. Do you have better images that could be presented, that show more consistent growth with your other assays?

I have switched out the images previously used in fig 4D for images taken from another one of the three replicates of this experiment. The brightness/contrast setting on these are more consistent with the others and it has both improved the general appearance and made it clear that the strains are behaving similarly to the other experiments, and the difference seen before was mostly visual. I do notice that the ΔlexA strain does not form as many colonies on the final dilution spot on the control plates in 4D as it did in 4A and 4B. I think this is because the person performing the experiments normalised the cultures to a lower OD value before plating and that it doesn’t alter the interpretation of figure 4D. We also see this effect in Figure 6F.

Reviewer 3 raised a number of other points that I believe can be largely addressed by clarifications in the writing. The data in Figure 6F is challenging to interpret because, as you note in the manuscript, the empty vector alone was deleterious specifically to this strain, an effect that was compounded by the presence of cipro. Moreover, the over-expression strain does not appear different from the empty vector strain. I suggest either trying the experiment as suggested by reviewer 3, or removing this result from the manuscript. As it stands, it is not conclusive. Finally, was there a reason that 6G did not include ∆lexA ∆higBA? If not, please include this strain.

The data in Figure 6F was from an older experiment that we had already obtained before submitting the original version of the paper. Since it included the conditions that had been requested by the reviewer, who considered the complementation an important condition to try, we decided to include it despite the ambiguous result. However, I agree that it does not support the main conclusion of the paper and the probable additive effects pointed out by Reviewer 3 complicate the interpretation. Therefore, I have taken it out.

The choice of strains in Figure 6G (new 6F) was because it was an experiment that had been performed some time ago, before we submitted this manuscript and received the reviewer comments, but after we had performed the mass spectrometry quantification which had showed us that levels of HigX protein were very similar in the ΔlexA single mutant and the ΔlexA Δ higBA double mutant (contrary to our initial expectations that the ΔlexA ΔhigBA double mutant would express much more). I agree it would have been optimal to include the double mutant in order to test for complementation properly, but at the time of doing the experiment we were mainly wanting to evaluate whether higX was epistatic to higBA in the ΔlexA background, which was why we prioritised the ΔlexA ΔhigX and ΔlexA ΔhigBAX strains here.

In reading this version, I noticed a few things that could improve the manuscript as well.

Figures 4A and 4b are blurry and pixelated compared to your other images, which are much crisper. Do you have better images from other replicates of these experiments? They are interpretable, thus it is not essential to replace them, but it would improve the aesthetic quality if you can.

I have inspected all the image files for 4A and B, but all replicates are unfortunately similar in quality to these ones. They were done at a time when we didn’t have access to a decent imager, which is why they have lower resolution and some reflection artefacts.

The abstract statement on line 28, is somewhat overstated. While higX may be conserved in alpha-proteobacteria, the genomic context does not seem to be conserved beyond the Caulobacteraceae. Figure S3 shows closely related species, which is valuable. But redoing the web flags analysis with the reduced database to capture more distant relatives does not support a conserved genomic context across the alpha-proteobacteria. Please revise the language.

We have rephrased this part of the abstract to “*higX* was conserved among many alpha-proteobacteria” which better reflects the observations without increasing the abstract length (new line 28)

Finally, I encourage you to consider revising your model figure to incorporate the idea that HigX-dependent phenotypes can be ascribed to filamentous cells, not exclusively to LexA.

I have added “(normal vs. filamentous)” to the figure annotation and extra text to the legend (new lines 961-62) to emphasise that the effects seen in filamentous cells are not LexA-dependent, but I have left the main figure as it is (with the Δ*lexA* labels above the filamentous cells) because we have not constructed higX deletions in any of the other filamentous strains so we can’t make this comparison for them yet.

***Response to Reviewers***

Reviewer #1: All the points raised by the reviewers have been addressed and new data requested by the reviewers have been incorporated in the revised manuscript, further supporting the main conclusions. In the present form, I do not have any other concern or request for this ms.

We thank reviewer 1 for their input, especially for the constructive suggestions for new experiments on the first version of the manuscript that we think have significantly enhanced the impact.

Reviewer #2: The manuscript by Brejndal et al. investigates an intriguing gene downstream of the higBA toxin-antitoxin pair that is involved in antibiotic resistance. The study stems from the confusing observation that deletion of higBA leads to a different phenotype than higB deletion. Through deletion and overexpression analysis, they find that the downstream higX gene is likely responsible for this drug resistance phenotype. In particular, they observe that HigX levels increase in ∆lexA cells and that this increase contributes to antibiotic sensitivity (but is not dependent on lexA!). Through ChIP-seq experiments, the authors also find that the HigX transcription factor targets a variety of envelope-related and metabolic genes, and that this targeting appears impaired in the ∆lexA background. Their genetic experiments and phylogenetic analyses also suggest that HigX acts independently of HigBA. Ultimately, the authors present a model in which elevated HigX reduce viability in associating with cell filamentation, potentially through a combination of transcriptional effects and membrane destabilization. Overall, I found the experimental logic to be sound and the manuscript to be well-written. Due to the variety of genetic strains and several screens, the interpretation is often complicated but presented clearly. The authors have adequately addressed my comments related to the original submission. I thank the authors for all the additional work they put into the manuscript. I have one minor comment that is related to new data added to the manuscript.

Minor comment:
Lines 254-255 and Fig. 4D: The authors note that the ∆lexA ∆sidA strain is more resistant to higX overexpression and ciprofloxacin, but the effect seems quite subtle in the figure (and hard to see). For example, is the ∆lexA ∆sidA strain really more resistant to higX overexpression than the ∆lexA strain? I’m not convinced based on the visible CFUs. Quantification, with clear reproducible differences, would better support the written conclusions. Alternatively, perhaps the authors can clarify their wording at these lines.

We thank reviewer 2 for their input to the manuscript. As described before, Figure 4D has been replaced with another replicate where the image quality is better. We agree that the *higX* overexpression effect is not strong in these strains, (although we did reproduce it in all 3 experiments) and have revised the text accordingly to “this strain was slightly more resistant to HigX overexpression than the Δ*lexA* mutant, although it was still sensitive to HigX overexpression combined with ciprofloxacin (Fig 4D)” (new lines 254-56).

Reviewer #3: I thank the authors for their additional work.
I find the new data more convincing and believe they support the hypothesis that HigX can impact cell envelope homeostasis in C. crescentus filamentous cells.
However, since heterologous complementation did not extend the role of HigX to other species, this effect appears to be restricted to Caulobacter crescentus. Therefore, the broader impact of the discovery seems limited and largely C. crescentus-centric.
I also have some reservations about the new data presented, which I detail below:

We thank reviewer 3 for their helpful input on the manuscript, please see below for our specific responses to the new comments.

Fig. 4D: Fig. 4D appears very different from the other plates shown in Fig. 4A, 6G or 7D, as though the strains did not grow properly on the control plate (compare 4A and 4D for the ∆lexA strains) and on the ciprofloxacin plates (compare 4A to 6G or 7D for the ∆lexA and ∆lexA∆higX strains). Do the authors have an additional replicate of Fig. 4D in which the strains behave similarly to those shown in Figs. 4A, 6G or 7D?

We have replaced the replicate used previously with one where the brightness/contrast settings are more consistent with the images used in the other figure panels, which now shows that the data are internally consistent and the differences seen in the older figure were primarily due to image settings (see also responses to Editors and to Reviewer 2).

Fig. 6E: Was vanillate used also to grow the strains carrying the empty vector? Based on the results obtained with both promoters affected by HigX overexpression, it appears that HigX functions as a repressor (i.e., overexpression of higX represses both PhigBA and PCCNA_03248). Do the quantitative proteomics data also support a repressor function?

All strains including controls are grown in the presence of gentamicin and vanillate, so that the medium conditions are identical for every strain in the experiment. We have added this information in the Materials and Methods (new line 658).

Our analysis of the quantitative proteomics data so far indicates that HigX can both activate and repress. With the caveat that the proteomics data will involve indirect as well as direct targets, we observed both enriched and depleted proteins in the data set when comparing ΔhigX to wild type, and the same when comparing vanillate-induced higX overexpression to empty vector.

Fig. 6F and 6G: Do all the plates contain vanillate, or only those carrying an overexpression construct? The legend says “Overexpression was induced by 50 μM vanillate”, does that mean that the plates with the empty vector does not contain vanillate? More generally, I find it unusual that the same background strain carrying the empty vector and the overexpression plasmid are not plated side by side on the same plate.

As described above, all plates contained vanillate. Regarding the strain arrangement, we chose this version over plating empty vector next to overexpression plasmid because the priority at the time of designing the experiment was to compare the two chromosomal genetic backgrounds (ΔlexA ΔhigX to ΔlexA ΔhigBAX) in the presence of the overexpression plasmid (see also Response to Editors).

Fig. 6F: Why does the strain carrying the empty pMT335 vector show reduced growth on ciprofloxacin compared with either the wild type or the same strain lacking a plasmid in Fig. 6D? It appears that the combination of gentamicin (used for plasmid maintenance) and ciprofloxacin may have additive deleterious effects, which complicates interpretation of the EOP assays. If the gentamicin is added in the agar medium, could it instead be added only to the liquid PYE used for preparing the serial dilutions?

We agree with this reviewer that this is likely due to confounding additive effects of the two antibiotics and have therefore taken this experiment out, as suggested by the editor.

Fig. 6G: I do not understand why the complementation was not performed in the same genetic background as the one used for the forward genetic screen and in Fig. 6D (ΔlexA ΔhigBA). If I understand correctly the hypothesis, the PamiC::himar1 insertion causes overexpression of amiC. Therefore, overexpression of amiC from pMT335 should mimic the himar1 insertion and restore ciprofloxacin resistance in the original ΔlexA ΔhigBA background. However, this strain is the only one absent from Fig. 6G.

This was largely a matter of timing, as described in the Response to Editors, since this data is from an early experiment that we had performed a couple of years earlier, before receiving the reviewer comments. We agree that it would have strengthened the argument if at the time we had included the ΔlexA ΔhigBA strain on the plate as well.

Line 454, Fig 7: I would word this part of the sentence (“with slightly reduced viability on control plates”) differently. The colonies formed are slightly smaller, but I do not think you can conclude reduced viability from this observation alone. Such a statement would require additional evidence, for example growth curves showing a growth delay. Especially because you did not observe the formation of smaller colonies when you overexpress higX in the single ∆lexA mutant (Fig. 4A).

In the new version of Figure 4D where the image quality is better than in 4A or the old version of 4D, we can see that we do observe smaller colonies on overexpression of higX in the ΔlexA background. However, we have replaced “viability” with “colony growth” here to avoid overstatement (new line 456)

Minor comments:
EOP: Efficiency of Plating: Please define the abbreviation the first time it is used (sorry if I miss it).

This has been included in the Materials and Methods subtitle (new line 651)

Fig. 5B and 5E: Since the CCNA_03217–CCNA_03218 intergenic region corresponds to your strongest ChIP-seq peak, why not test whether PCCNA_03217 is affected by higX overexpression?

We did construct and test this reporter, and higX overexpression did not do anything (see below)

Fig. 7: What is the percentage identity (and/or similarity) between C. crescentus HigX and the HigX homologs from C. segnis and B. lenta? Have you tried to detect them with your HigX antibody? Perhaps the lack of complementation is due to poor protein production or stability.

The C segnis HigX has 68% identity/76% similarity with HigX from NA1000, and the B. lenta HigX has 46% identity/57% similarity with HigX from NA1000. These values have been added to the manuscript in the text (new lines 452-454). We have not tried detection of these with the HigX antibody yet, mostly due to lack of time, but we anticipate that it wouldn’t detect the two heterologous HigXs equally because the peptide epitope used for raising the antibody is conserved 100% in C. segnis, but not in B. lenta. We agree that heterologous protein stability or production is an important factor to consider in future studies.

Fig. S2: It would be helpful to indicate the strain background in which the ChIP-seq was performed like in fig 5A.

We have annotated the line plot in figure S2 to indicate this

These points should be addressed. I am particularly puzzled by the apparent differences in the spot dilution assays, which look quite different despite representing similar conditions and strains.

I hope that the use of a different replicate with better image quality in Figure 4D will resolve this concern.
