# Supplementary material for: An ancestral transmembrane transcription factor couples cell envelope regulation and the SOS response in Caulobacter crescentus
Source: PLoS Genet. 2026 Jul 15;22(7):e1011986. doi: 10.1371/journal.pgen.1011986 (PMC13384398; doi:10.1371/journal.pgen.1011986)
Supplement: Peer review history

# Peer review: Decision Letter 0

**DOI:** 10.1371/journal.pgen.1011986.r001

**Authors:** Danielle A. Garsin, Aretha Fiebig

6 Jan 2026

PGENETICS-D-25-01322

An ancestral transmembrane transcription factor couples cell envelope regulation and the SOS response in Caulobacter crescentus

PLOS Genetics

Dear Dr. Kirkpatrick,

Thank you for submitting your manuscript to PLOS Genetics. After careful consideration, we feel that it has merit but does not fully meet PLOS Genetics's publication criteria as it currently stands. Therefore, we invite you to submit a revised version of the manuscript that addresses the points raised during the review process.

Please submit your revised manuscript by Mar 07 2026 11:59PM. If you will need more time than this to complete your revisions, please reply to this message or contact the journal office at plosgenetics@plos.org. When you're ready to submit your revision, log on to https://www.editorialmanager.com/pgenetics/ and select the 'Submissions Needing Revision' folder to locate your manuscript file.

Please include the following items when submitting your revised manuscript:

* A letter that responds to each point raised by the editor and reviewer(s). You should upload this letter as a separate file labeled 'Response to Reviewers'. This file does not need to include responses to any formatting updates and technical items listed in the 'Journal Requirements' section below.

* A marked-up copy of your manuscript that highlights changes made to the original version. You should upload this as a separate file labeled 'Revised Manuscript with Track Changes'.

* An unmarked version of your revised paper without tracked changes. You should upload this as a separate file labeled 'Manuscript'.

If you would like to make changes to your financial disclosure, competing interests statement, or data availability statement, please make these updates within the submission form at the time of resubmission. Guidelines for resubmitting your figure files are available below the reviewer comments at the end of this letter.

We look forward to receiving your revised manuscript.

Kind regards,

Aretha Fiebig, PhD

Academic Editor

PLOS Genetics

Danielle Garsin

Section Editor

PLOS Genetics

Aimée Dudley

Editor-in-Chief

PLOS Genetics

Anne Goriely

Editor-in-Chief

PLOS Genetics

**Additional Editor Comments:**

Thank you for submitting this work to PLOS Genetics. The reviewers of this manuscript were all enthusiastic about the quality of the experiments presented and the potential impact of the work. However, reviewers expressed that some features of the model should be better supported, specifically why HigX phenotypes require deletion of lexA and whether HigX binding to DNA has regulatory consequences on the downstream (envelope related) genes.

**Journal Requirements:**

1) Please ensure that the CRediT author contributions listed for every co-author are completed accurately and in full.

At this stage, the following Authors/Authors require contributions: Kamilla Ankær Brejndal, Nikolaj Vestergaard Hansen, Koyel Ghosh, Sebastian Nielsen, Lykke Haastrup Hansen, Lene A Jakobsen, Martin R Larsen, and Clare Louise Kirkpatrick. Please ensure that the full contributions of each author are acknowledged in the "Add/Edit/Remove Authors" section of our submission form.

The list of CRediT author contributions may be found here: https://journals.plos.org/plosgenetics/s/authorship#loc-author-contributions

2) Please upload all main figures as separate Figure files in .tif or .eps format. For more information about how to convert and format your figure files please see our guidelines:

https://journals.plos.org/plosgenetics/s/figures

3) Some material included in your submission may be copyrighted. According to PLOSu2019s copyright policy, authors who use figures or other material (e.g., graphics, clipart, maps) from another author or copyright holder must demonstrate or obtain permission to publish this material under the Creative Commons Attribution 4.0 International (CC BY 4.0) License used by PLOS journals. Please closely review the details of PLOSu2019s copyright requirements here: PLOS Licenses and Copyright. If you need to request permissions from a copyright holder, you may use PLOS's Copyright Content Permission form.

Please respond directly to this email and provide any known details concerning your material's license terms and permissions required for reuse, even if you have not yet obtained copyright permissions or are unsure of your material's copyright compatibility. Once you have responded and addressed all other outstanding technical requirements, you may resubmit your manuscript within Editorial Manager.

Potential Copyright Issues:

- Figure 6. Please confirm whether you drew the images / clip-art within the figure panels by hand. If you did not draw the images, please provide (a) a link to the source of the images or icons and their license / terms of use; or (b) written permission from the copyright holder to publish the images or icons under our CC BY 4.0 license. Alternatively, you may replace the images with open source alternatives. See these open source resources you may use to replace images / clip-art:

- https://commons.wikimedia.org

- https://openclipart.org/.

**Reviewers' comments:**

Reviewer's Responses to Questions

**Comments to the Authors:**

**Please note here if the review is uploaded as an attachment.**

Reviewer #1: In this manuscript, the authors characterised the enigmatic membrane-associated transcription factor called HigX in Caulobacter crescentus. In C. crescentus, higX is encoded downstream of the TA system higBA, whose expression is repressed by LexA and the HigA antitoxin. While a previous study showed that ∆lexA cells display a HigB-dependent hypersensitivity to ciprofloxacin, the authors show in this manuscript that the concomitant inactivation of both repressors LexA and HigA leads to a HigB-independent hypersensitivity to ciprofloxacin, which depends on HigX. The expression of higX from an inducible promoter on a multi-copy plasmid also leads to ciprofloxacin hypersensitivity but only in a ∆lexA background. Interestingly, ChIP-seq experiments show that HigX binds to promoter regions of cell envelope-associated genes and that DNA binding of HigX requires LexA. In addition, mutations in cell envelope-related genes were found in forward genetic screens to suppress HigX-dependent sensitivity to ciprofloxacin in a ∆lexA ∆higBA background, suggesting that overproduction of HigX leads to destabilization of the cell envelope. Finally, a thorough phylogenetic analysis and RSCU of the hig locus show that higX is widespread in Caulobacterales whereas higBA was likely acquired by horizontal gene transfer in the Caulobacter genus.

Although the phenotypes displayed by HigX overexpression are interesting, they are only observed in ∆lexA backgrounds and upon antibiotics treatment, limiting the understanding of its biological role.

In particular, we don’t know why the phenotypes are observed only in ∆lexA, even when higX is overproduced from an inducible promoter on a multicopy plasmid. In fact, it is unclear if the toxicity of HigX in ∆lexA cells exposed to ciprofloxacin comes from transcriptional misregulation, since HigX cannot bind anymore to DNA in the absence of lexA, or from the (over)accumulation of HigX in the membrane of filamentous cells.

I would therefore strongly encourage the authors to further characterise the dependency of the HigX-dependent phenotypes on LexA.

Here are suggestions to address this question:

- The authors proposed that the extreme filamentation of ∆lexA cells leads to excessive accumulation of HigX in the cell envelope, which in turn weakens its integrity and facilitates access of ciprofloxacin to the cytoplasm. If this is true, inactivating sidA in ∆lexA should decreased ciprofloxacin sensitivity upon higX overexpression since ∆lexA∆sidA cells do not filament (DOI: 10.1101/gad.2038911).

- On the other hand, viable or conditional mutants that generate filamentous cells could be used to test whether cellular filamentation indeed sensitises to ciprofloxacin or cephalexin upon higX overexpression. There are indeed numerous filamentous cell division or cell cycle mutants available in Caulobacter.

- As a complementary approach to the higX-noTM variant, a higX-onlyTM could be expressed from the pMT335 plasmid vector to test if the TM domains alone can induce the same hypersensitivity to ciprofloxacin. If it works, it would support the hypothesis of cell envelope loading.

- To test whether DNA binding of HigX is responsible for the phenotypes, the HigX-dependent regulon should be determined by ChIP-seq (and RNA-seq) in cells exposed to ciprofloxacin. Indeed, the ChIP-seq has been done in PYE, a condition in which neither the lexA nor the hig mutants impact growth. To avoid cell death with ciprofloxacin, one of the suppressors could be used. For instance, WT, ∆lexA ∆higBA, ∆lexA ∆higBA 03248::himar1 and ∆lexA ∆higBAX strains could be grown in the presence of the antibiotic before performing the ChIP-seq or extracting RNA.

If the first three experiments support the first hypothesis, there is even no need to address the second hypothesis.

Minor comments

- L223: It is stated that the abundance of HigX peptides was “strongly depleted in ∆higX”.

Why HigX peptides are still detected in mass spectrometry in ∆higX cells?

- Do the suppressors also induce ciprofloxacin resistance to ∆lexA mutant? If so, this would mean that the suppressors also protect from HigB-dependent toxicity.

Reviewer #2: Uploaded as an attachment

Reviewer #3: The review is uploaded as an attachment.

**********

**Have all data underlying the figures and results presented in the manuscript been provided?**

Large-scale datasets should be made available via a public repository as described in the *PLOS Genetics*  data availability policy, and numerical data that underlies graphs or summary statistics should be provided in spreadsheet form as supporting information.

Reviewer #1: Yes

Reviewer #2: Yes

Reviewer #3: Yes

**********

PLOS authors have the option to publish the peer review history of their article (what does this mean?). If published, this will include your full peer review and any attached files.

If you choose “no”, your identity will remain anonymous but your review may still be made public.

**Do you want your identity to be public for this peer review?**  For information about this choice, including consent withdrawal, please see our Privacy Policy.

Reviewer #1: No

Reviewer #2: No

Reviewer #3: No

[NOTE: If reviewer comments were submitted as an attachment file, they will be attached to this email and accessible via the submission site. Please log into your account, locate the manuscript record, and check for the action link "View Attachments". If this link does not appear, there are no attachment files.]

**Figure resubmission:**

While revising your submission, we strongly recommend that you use PLOS’s NAAS tool (https://ngplosjournals.pagemajik.ai/artanalysis) to test your figure files. NAAS can convert your figure files to the TIFF file type and meet basic requirements (such as print size, resolution), or provide you with a report on issues that do not meet our requirements and that NAAS cannot fix.

After uploading your figures to PLOS’s NAAS tool - https://ngplosjournals.pagemajik.ai/artanalysis, NAAS will process the files provided and display the results in the "Uploaded Files" section of the page as the processing is complete. If the uploaded figures meet our requirements (or NAAS is able to fix the files to meet our requirements), the figure will be marked as "fixed" above. If NAAS is unable to fix the files, a red "failed" label will appear above. When NAAS has confirmed that the figure files meet our requirements, please download the file via the download option, and include these NAAS processed figure files when submitting your revised manuscript.

**Reproducibility:**

To enhance the reproducibility of your results, we recommend that authors of applicable studies deposit laboratory protocols in protocols.io, where a protocol can be assigned its own identifier (DOI) such that it can be cited independently in the future. Additionally, PLOS ONE offers an option to publish peer-reviewed clinical study protocols. Read more information on sharing protocols at https://plos.org/protocols?utm_medium=editorial-email&utm_source=authorletters&utm_campaign=protocols

Attachment Submitted filename: Brejndaletal2025.docx

Attachment Submitted filename: PLOS Genetics 2025 Brejndal.docx

# Author comment: Author response to Decision Letter 1

**DOI:** 10.1371/journal.pgen.1011986.r002

29 May 2026

Attachment Submitted filename: Response to Reviewers.docx

# Peer review: Decision Letter 1

**DOI:** 10.1371/journal.pgen.1011986.r003

**Authors:** Danielle A. Garsin, Aretha Fiebig

22 Jun 2026

PGENETICS-D-25-01322R1

An ancestral transmembrane transcription factor couples cell envelope regulation and the SOS response in Caulobacter crescentus

PLOS Genetics

Dear Dr. Kirkpatrick,

Thank you for submitting your manuscript to PLOS Genetics. After careful consideration, we feel that it has merit but does not fully meet PLOS Genetics's publication criteria as it currently stands. Therefore, we invite you to submit a revised version of the manuscript that addresses the points raised during the review process.

Please submit your revised manuscript within by Jul 22 2026 11:59PM. If you will need more time than this to complete your revisions, please reply to this message or contact the journal office at plosgenetics@plos.org. When you're ready to submit your revision, log on to https://www.editorialmanager.com/pgenetics/ and select the 'Submissions Needing Revision' folder to locate your manuscript file.

Please include the following items when submitting your revised manuscript:

* A letter that responds to each point raised by the editor and reviewer(s). You should upload this letter as a separate file labeled 'Response to Reviewers'. This file does not need to include responses to formatting updates and technical items listed in the 'Journal Requirements' section below.

* A marked-up copy of your manuscript that highlights changes made to the original version. You should upload this as a separate file labeled 'Revised Manuscript with Track Changes'.

* An unmarked version of your revised paper without tracked changes. You should upload this as a separate file labeled 'Manuscript'.

If you would like to make changes to your financial disclosure, competing interests statement, or data availability statement, please make these updates within the submission form at the time of resubmission. Guidelines for resubmitting your figure files are available below the reviewer comments at the end of this letter.

As the corresponding author, your ORCID iD is verified in the submission system and will appear in the published article. PLOS supports the use of ORCID, and we encourage all coauthors to register for an ORCID iD and use it as well. Please encourage your coauthors to verify their ORCID iD within the submission system before final acceptance, as unverified ORCID iDs will not appear in the published article. Only the individual author can complete the verification step; PLOS staff cannot verify ORCID iDs on behalf of authors.

We look forward to receiving your revised manuscript.

Kind regards,

Aretha Fiebig, PhD

Academic Editor

PLOS Genetics

Danielle Garsin

Section Editor

PLOS Genetics

Aimée Dudley

Editor-in-Chief

PLOS Genetics

Anne Goriely

Editor-in-Chief

PLOS Genetics

**Additional Editor Comments (if provided):**

Thank you for the major revisions and additional work in your manuscript regarding the function of higX. The initial concerns of the reviewers were largely satisfied. The new data with other filaments mutants and with the ∆lexA ∆sidA strain nicely strengthens this work by clarifying models about higX activity. However, some concerns were raised about the new data that require minor revisions.

Multiple reviews raised concerns about the variability in the spot dilution assays presented, particularly in 4D. In addition, the images in 4D seem poorly illuminated and difficult to interpret. Do you have better images that could be presented, that show more consistent growth with your other assays?

Reviewer 3 raised a number of other points that I believe can be largely addressed by clarifications in the writing. The data in Figure 6F is challenging to interpret because, as you note in the manuscript, the empty vector alone was deleterious specifically to this strain, an effect that was compounded by the presence of cipro. Moreover, the over-expression strain does not appear different from the empty vector strain. I suggest either trying the experiment as suggested by reviewer 3, or removing this result from the manuscript. As it stands, it is not conclusive. Finally, was there a reason that 6G did not include ∆lexA ∆higBA? If not, please include this strain.

In reading this version, I noticed a few things that could improve the manuscript as well.

Figures 4A and 4b are blurry and pixelated compared to your other images, which are much crisper. Do you have better images from other replicates of these experiments? They are interpretable, thus it is not essential to replace them, but it would improve the aesthetic quality if you can.

The abstract statement on line 28, is somewhat overstated. While higX may be conserved in alpha-proteobacteria, the genomic context does not seem to be conserved beyond the Caulobacteraceae. Figure S3 shows closely related species, which is valuable. But redoing the web flags analysis with the reduced database to capture more distant relatives does not support a conserved genomic context across the alpha-proteobacteria. Please revise the language.

Finally, I encourage you to consider revising your model figure to incorporate the idea that HigX-dependent phenotypes can be ascribed to filamentous cells, not exclusively to LexA.

**Reviewers' comments:**

Reviewer's Responses to Questions

**Comments to the Authors:**

**Please note here if the review is uploaded as an attachment.**

Reviewer #1: All the points raised by the reviewers have been addressed and new data requested by the reviewers have been incorporated in the revised manuscript, further supporting the main conclusions. In the present form, I do not have any other concern or request for this ms.

Reviewer #2: The manuscript by Brejndal et al. investigates an intriguing gene downstream of the higBA toxin-antitoxin pair that is involved in antibiotic resistance. The study stems from the confusing observation that deletion of higBA leads to a different phenotype than higB deletion. Through deletion and overexpression analysis, they find that the downstream higX gene is likely responsible for this drug resistance phenotype. In particular, they observe that HigX levels increase in ∆lexA cells and that this increase contributes to antibiotic sensitivity (but is not dependent on lexA!). Through ChIP-seq experiments, the authors also find that the HigX transcription factor targets a variety of envelope-related and metabolic genes, and that this targeting appears impaired in the ∆lexA background. Their genetic experiments and phylogenetic analyses also suggest that HigX acts independently of HigBA. Ultimately, the authors present a model in which elevated HigX reduce viability in associating with cell filamentation, potentially through a combination of transcriptional effects and membrane destabilization. Overall, I found the experimental logic to be sound and the manuscript to be well-written. Due to the variety of genetic strains and several screens, the interpretation is often complicated but presented clearly. The authors have adequately addressed my comments related to the original submission. I thank the authors for all the additional work they put into the manuscript. I have one minor comment that is related to new data added to the manuscript.

Minor comment:

Lines 254-255 and Fig. 4D: The authors note that the ∆lexA ∆sidA strain is more resistant to higX overexpression and ciprofloxacin, but the effect seems quite subtle in the figure (and hard to see). For example, is the ∆lexA ∆sidA strain really more resistant to higX overexpression than the ∆lexA strain? I’m not convinced based on the visible CFUs. Quantification, with clear reproducible differences, would better support the written conclusions. Alternatively, perhaps the authors can clarify their wording at these lines.

Reviewer #3: I thank the authors for their additional work.

I find the new data more convincing and believe they support the hypothesis that HigX can impact cell envelope homeostasis in C. crescentus filamentous cells.

However, since heterologous complementation did not extend the role of HigX to other species, this effect appears to be restricted to Caulobacter crescentus. Therefore, the broader impact of the discovery seems limited and largely C. crescentus-centric.

I also have some reservations about the new data presented, which I detail below:

Fig. 4D: Fig. 4D appears very different from the other plates shown in Fig. 4A, 6G or 7D, as though the strains did not grow properly on the control plate (compare 4A and 4D for the ∆lexA strains) and on the ciprofloxacin plates (compare 4A to 6G or 7D for the ∆lexA and ∆lexA∆higX strains). Do the authors have an additional replicate of Fig. 4D in which the strains behave similarly to those shown in Figs. 4A, 6G or 7D?

Fig. 6E: Was vanillate used also to grow the strains carrying the empty vector? Based on the results obtained with both promoters affected by HigX overexpression, it appears that HigX functions as a repressor (i.e., overexpression of higX represses both PhigBA and PCCNA_03248). Do the quantitative proteomics data also support a repressor function?

Fig. 6F and 6G: Do all the plates contain vanillate, or only those carrying an overexpression construct? The legend says “Overexpression was induced by 50 μM vanillate”, does that mean that the plates with the empty vector does not contain vanillate? More generally, I find it unusual that the same background strain carrying the empty vector and the overexpression plasmid are not plated side by side on the same plate.

Fig. 6F: Why does the strain carrying the empty pMT335 vector show reduced growth on ciprofloxacin compared with either the wild type or the same strain lacking a plasmid in Fig. 6D? It appears that the combination of gentamicin (used for plasmid maintenance) and ciprofloxacin may have additive deleterious effects, which complicates interpretation of the EOP assays. If the gentamicin is added in the agar medium, could it instead be added only to the liquid PYE used for preparing the serial dilutions?

Fig. 6G: I do not understand why the complementation was not performed in the same genetic background as the one used for the forward genetic screen and in Fig. 6D (ΔlexA ΔhigBA). If I understand correctly the hypothesis, the PamiC::himar1 insertion causes overexpression of amiC. Therefore, overexpression of amiC from pMT335 should mimic the himar1 insertion and restore ciprofloxacin resistance in the original ΔlexA ΔhigBA background. However, this strain is the only one absent from Fig. 6G.

Line 454, Fig 7: I would word this part of the sentence (“with slightly reduced viability on control plates”) differently. The colonies formed are slightly smaller, but I do not think you can conclude reduced viability from this observation alone. Such a statement would require additional evidence, for example growth curves showing a growth delay. Especially because you did not observe the formation of smaller colonies when you overexpress higX in the single ∆lexA mutant (Fig. 4A).

Minor comments:

EOP: Efficiency of Plating: Please define the abbreviation the first time it is used (sorry if I miss it).

Fig. 5B and 5E: Since the CCNA_03217–CCNA_03218 intergenic region corresponds to your strongest ChIP-seq peak, why not test whether PCCNA_03217 is affected by higX overexpression?

Fig. 7: What is the percentage identity (and/or similarity) between C. crescentus HigX and the HigX homologs from C. segnis and B. lenta? Have you tried to detect them with your HigX antibody? Perhaps the lack of complementation is due to poor protein production or stability.

Fig. S2: It would be helpful to indicate the strain background in which the ChIP-seq was performed like in fig 5A.

These points should be addressed. I am particularly puzzled by the apparent differences in the spot dilution assays, which look quite different despite representing similar conditions and strains.

**********

**Have all data underlying the figures and results presented in the manuscript been provided?**

Large-scale datasets should be made available via a public repository as described in the *PLOS Genetics*  data availability policy, and numerical data that underlies graphs or summary statistics should be provided in spreadsheet form as supporting information.

Reviewer #1: Yes

Reviewer #2: Yes

Reviewer #3: Yes

**********

PLOS authors have the option to publish the peer review history of their article (what does this mean?). If published, this will include your full peer review and any attached files.

If you choose “no”, your identity will remain anonymous but your review may still be made public.

**Do you want your identity to be public for this peer review?**  For information about this choice, including consent withdrawal, please see our Privacy Policy.

Reviewer #1: No

Reviewer #2: No

Reviewer #3: No

[NOTE: If reviewer comments were submitted as an attachment file, they will be attached to this email and accessible via the submission site. Please log into your account, locate the manuscript record, and check for the action link "View Attachments". If this link does not appear, there are no attachment files.]

**Figure resubmission:**

While revising your submission, we strongly recommend that you use PLOS’s NAAS tool (https://ngplosjournals.pagemajik.ai/artanalysis) to test your figure files. NAAS can convert your figure files to the TIFF file type and meet basic requirements (such as print size, resolution), or provide you with a report on issues that do not meet our requirements and that NAAS cannot fix.

After uploading your figures to PLOS’s NAAS tool - https://ngplosjournals.pagemajik.ai/artanalysis, NAAS will process the files provided and display the results in the "Uploaded Files" section of the page as the processing is complete. If the uploaded figures meet our requirements (or NAAS is able to fix the files to meet our requirements), the figure will be marked as "fixed" above. If NAAS is unable to fix the files, a red "failed" label will appear above. When NAAS has confirmed that the figure files meet our requirements, please download the file via the download option, and include these NAAS processed figure files when submitting your revised manuscript.

**Reproducibility:**

To enhance the reproducibility of your results, we recommend that authors deposit laboratory protocols in protocols.io, where a protocol can be assigned its own identifier (DOI) such that it can be cited independently in the future. Additionally, PLOS ONE offers an option to publish peer-reviewed clinical study protocols. Read more information on sharing protocols at https://plos.org/protocols?utm_medium=editorial-email&utm_source=authorletters&utm_campaign=protocols

# Author comment: Author response to Decision Letter 2

**DOI:** 10.1371/journal.pgen.1011986.r004

26 Jun 2026

Attachment Submitted filename: Response to Reviewers 2.docx

# Editor report: Decision Letter 2

**DOI:** 10.1371/journal.pgen.1011986.r005

**Authors:** Danielle A. Garsin, Aretha Fiebig

1 Jul 2026

Dear Dr Kirkpatrick,

We are pleased to inform you that your manuscript entitled "An ancestral transmembrane transcription factor couples cell envelope regulation and the SOS response in Caulobacter crescentus" has been editorially accepted for publication in PLOS Genetics. Congratulations!

Before your submission can be formally accepted and sent to production you will need to complete our formatting changes, which you will receive in a follow up email. Please be aware that it may take several days for you to receive this email; during this time no action is required by you. Please note: the accept date on your published article will reflect the date of this provisional acceptance, but your manuscript will not be scheduled for publication until the required changes have been made.

Once your paper is formally accepted, an uncorrected proof of your manuscript will be published online ahead of the final version, unless you’ve already opted out via the online submission form. If, for any reason, you do not want an earlier version of your manuscript published online or are unsure if you have already indicated as such, please let the journal staff know immediately at plosgenetics@plos.org.

In the meantime, please log into Editorial Manager at https://www.editorialmanager.com/pgenetics/, click the "Update My Information" link at the top of the page, and update your user information to ensure an efficient production and billing process. Note that PLOS requires an ORCID iD for all corresponding authors. Therefore, please ensure that you have an ORCID iD and that it is validated in Editorial Manager. To do this, go to ‘Update my Information’ (in the upper left-hand corner of the main menu), and click on the Fetch/Validate link next to the ORCID field.  This will take you to the ORCID site and allow you to create a new iD or authenticate a pre-existing iD in Editorial Manager.

If you have a press-related query, or would like to know about making your underlying data available (as you will be aware, this is required for publication), please see the end of this email. If your institution or institutions have a press office, please notify them about your upcoming article at this point, to enable them to help maximise its impact. Inform journal staff as soon as possible if you are preparing a press release for your article and need a publication date.

Thank you again for supporting open-access publishing; we are looking forward to publishing your work in PLOS Genetics!

Yours sincerely,

Aretha Fiebig, PhD

Academic Editor

PLOS Genetics

Danielle Garsin

Section Editor

PLOS Genetics

Aimée Dudley

Editor-in-Chief

PLOS Genetics

Anne Goriely

Editor-in-Chief

PLOS Genetics

www.plosgenetics.org

BlueSky: @plos.bsky.social

----------------------------------------------------

Comments from the reviewers (if applicable):

Thank you for your careful attention to the reviewers feedback. This work provides an important characterization of the novel factor, higX, and its role envelope regulation. Thank you for this work.

----------------------------------------------------

**Data Deposition**

If you have submitted a Research Article or Front Matter that has associated data that are not suitable for deposition in a subject-specific public repository (such as GenBank or ArrayExpress), one way to make that data available is to deposit it in the Dryad Digital Repository. As you may recall, we ask all authors to agree to make data available; this is one way to achieve that. A full list of recommended repositories can be found on our website.

The following link will take you to the Dryad record for your article, so you won't have to re‐enter its bibliographic information, and can upload your files directly:

http://datadryad.org/submit?journalID=pgenetics&manu=PGENETICS-D-25-01322R2

More information about depositing data in Dryad is available at http://www.datadryad.org/depositing. If you experience any difficulties in submitting your data, please contact help@datadryad.org for support.

Additionally, please be aware that our data availability policy requires that all numerical data underlying display items are included with the submission, and you will need to provide this before we can formally accept your manuscript, if not already present.

----------------------------------------------------

**Press Queries**

If you or your institution will be preparing press materials for this manuscript, or if you need to know your paper's publication date for media purposes, please inform the journal staff as soon as possible so that your submission can be scheduled accordingly. Your manuscript will remain under a strict press embargo until the publication date and time. This means an early version of your manuscript will not be published ahead of your final version. PLOS Genetics may also choose to issue a press release for your article. If there's anything the journal should know or you'd like more information, please get in touch via plosgenetics@plos.org.

# Editor report: Acceptance letter

**DOI:** 10.1371/journal.pgen.1011986.r006

**Authors:** Danielle A. Garsin, Aretha Fiebig

PGENETICS-D-25-01322R2

An ancestral transmembrane transcription factor couples cell envelope regulation and the SOS response in Caulobacter crescentus

Dear Dr Kirkpatrick,

We are pleased to inform you that your manuscript entitled "

An ancestral transmembrane transcription factor couples cell envelope regulation and the SOS response in Caulobacter crescentus" has been formally accepted for publication in PLOS Genetics! Your manuscript is now with our production department and you will be notified of the publication date in due course.

The corresponding author will soon be receiving a typeset proof for review, to ensure errors have not been introduced during production. Please review the PDF proof of your manuscript carefully, as this is the last chance to correct any errors. Please note that major changes, or those which affect the scientific understanding of the work, will likely cause delays to the publication date of your manuscript.

Soon after your final files are uploaded, unless you have opted out or your manuscript is a front-matter piece, the early version of your manuscript will be published online. The date of the early version will be your article's publication date. The final article will be published to the same URL, and all versions of the paper will be accessible to readers.

For Research Articles, you will receive an invoice from PLOS for your publication fee after your manuscript has reached the completed accept phase. If you receive an email requesting payment before acceptance or for any other service, this may be a phishing scheme. Learn how to identify phishing emails and protect your accounts at https://explore.plos.org/phishing.

Thank you again for supporting PLOS Genetics and open-access publishing. We are looking forward to publishing your work!

With kind regards,

Kannan R K Kuppusamy, B.TECH BIOTECHNOLOGY

PLOS Genetics

On behalf of:

The PLOS Genetics Team

Carlyle House, Carlyle Road, Cambridge CB4 3DN | United Kingdom

plosgenetics@plos.org | +44 (0) 1223-442823

plosgenetics.org | Twitter: @PLOSGenetics
